# Computational and experimental engineering of a *Pleurotus citrinopileatus* lipases: Structural insights and functional optimization to adapt the hydrolytic profile for cheese applications

**DOI:** 10.1016/j.fochx.2026.103639

**Published:** 2026-02-06

**Authors:** Lea Henrich, Niklas Broel, Jonathan Schüler, Marius Lang, Binglin Li, Martin Gand

**Affiliations:** a Institute of Food Chemistry and Food Biotechnology, Justus Liebig University Giessen, Germany; b Institute of Food Science and Technology, Chinese Academy of Agricultural Sciences, Beijing, China; c College of Food Science and Engineering, Northwest University, Shaanxi, China; d Cangzhou Academy of Agriculture and Forestry Sciences, Cangzhou, China

**Keywords:** Fungal lipase, *Pleurotus citrinopileatus*, Enzyme engineering, Semi-rational design, Cheese making, Chain length specificity, Free fatty acids

## Abstract

Mutants of a golden oyster mushroom lipase (*Pleurotus citrinopileatus*; PCI_Lip), were engineered to enhance hydrolysis profiles for cheese production. Key residues affecting activity were identified by SMME (Structure model based on AlphaFold3 prediction, followed by Molecular docking, Molecular dynamics, and Experimental validation) in three rounds of mutagenesis. Double mutants S163M + L302G and L302G + L305A showed significant improvements in photometric assays. The pNPH/pNPP ratios of 11.6 ± 1.1 and 10.4 ± 1.1 of both mutants, respectively, were improved compared to the wild type's 0.4 ± 0.1. Cheese made with S163M + L302G had a taste similar to the one prepared with a commercial enzyme in a descriptive testing, and both mutants demonstrated enhanced catalytic efficiency for short- to medium-chain fatty acids. These findings could be further confirmed by SPME-GC–MS analysis of the cheese samples. A structurally similar lipase from *Phlebia centrifuga* was investigated due to structural similarity with RMSD of 0.444 Å. The comparison of both lipases highlighted the key role of the lid domain in substrate specificity and affinity. This work advances the understanding of fungal lipases and their potential in food biotechnology.

## Highlights

• Basidiomycota lipase libraries with optimized hydrolysis profiles were investigated as alternatives to pregastric esterases
• Comparative analysis of the lipases provided deeper insights into the role of the lid domain
• Protein engineering was applied to tailor PCI_Lip to cheese-making requirements
• Key residues influencing hydrolytic activity were identified and characterized

## 1 Introduction

Fungi represent a vast but largely untapped source of enzymes, particularly within the phylum Basidiomycota. Their secretome, which comprises all secreted enzymes, adapts dynamically to environmental conditions such as carbon and nitrogen availability, making them highly versatile biocatalysts (Bouws et al., 2008; Filiatrault-Chastel et al., 2021). As the shift toward renewable biological resources accelerates, fungal enzymes are gaining importance in white biotechnology, particularly for biopolymer degradation and fine chemical synthesis. Currently, most applied enzymes from fungal origin in white biotechnology are derived from Ascomycota (El-Gendi et al., 2021) while Basidiomycota, comprising more than 30,000 species remain underexplored (Berger & Ersoy, 2022). There is special interest of industry in esterases that are capable of catalyzing the hydrolysis of triglycerides into glycerol and free fatty acids (FFA) and the formation of mono- and diglycerides, commonly named lipases (EC 3.1.1.3). Those enzymes are the key players in enzyme-modified cheese (EMC), which is widely produced by the food industry to efficiently enhance cheese flavor in a cost-effective manner. By accelerating the natural ripening process through enzymatic application, EMC achieves more intense flavors by releasing free fatty acids (FFAs). Short-chain FFAs are particularly important for imparting the sharp, tangy, and savory notes characteristic of cheese types such as Provolone and Feta. Medium-chain FFAs contribute to creamy and slightly fatty flavors, while long-chain FFAs on the one hand enhance the buttery mouthfeel and add a waxy texture, enriching the overall sensory complexity of cheese, while on the other hand if their concentration is too high causing soapy off-flavors (Ebba et al., 2012; Efthymiou, 1967; Hanselman et al., 2021; Yoo & Lee, 2024). Still the most commonly used traditional lipolytic enzymes originate from various animal sources, including native milk enzymes, rennet paste, and pregastric esterases (PGEs) (Collins et al., 2003; Wilkinson, Doolan, & Kilcawley, 2011). PGEs, secreted by the sublingual glands of milk-drinking animals like goat kids and lambs, preferentially hydrolyze short to medium chain fatty acids (C4:0 – C10:0), which give a savory flavor in the final product (Fox & Wallace, 1997)(Moschopoulou, 2011). Most commercially available lipases, such as Piccantase A from *Rhizomucor miehei*, exhibit broad substrate specificity, hydrolyzing triacylglycerides indiscriminately. This enhances the overall cheese flavor, but due to the non-selective release of FFAs, ultimately affecting the balance and refinement of the cheese's sensory.

Traditional animal-derived lipase preparations are unsuitable for vegetarian diets and may not comply with kosher or halal dietary requirements, limiting their marketability amid the rising demand for ethical and dietary-compliant alternatives. This could be partially demonstrated by the global market value of 65 billion USD of halal ingredients was increased with 6% compound annual growth rate from 2019 to 2025 (Markets and Markets, 2019). Mushrooms from the Basidiomycota phylum naturally meet these dietary requirements and have demonstrated promising enzymatic activities, as revealed by fungal secretome studies over the past decades. A promising candidate was identified in a study by Sowa et al., where the purified lipase from the golden oyster mushroom, *Pleurotus citrinopileatus* (PCI_Lip), was used to produce cheese with sensory properties resembling those made with the PGE.

However, a slightly soapy aftertaste remained (Sowa et al., 2022). This aftertaste attributed to an enhanced release of long chain fatty acids (LCFA) is considered as an off-flavor in cheese. Therefore, the amount of LCFA should be kept low. Depending on the cheese variety the ratio of short chain fatty acids (SCFA) to LCFA should be between 0.4 and 0.7 (Delgado et al., 2011; Rani & Jagtap, 2019). To address this issue, Broel et al. successfully applied a semi-rational design strategy, supported by biomolecular simulations, to engineer site-directed mutants with improved selectivity. Their work led to enhancements in the hydrolysis activity and substrate selectivity of certain variants, contributing to a more refined cheese flavor profile. Nevertheless, a substantial amount of long-chain fatty acids was released by hydrolysis, contributing to the undesirable soapy aftertaste. Additionally, the low expression levels of PCI_Lip further limited the enzyme's potential for broader application (Broel et al., 2022).

To overcome the limitations of site-directed point mutations in improving substrate specificity, a multiple-site mutation approach, similarity like the SMME, was explored in this work. Unlike single-point mutations, which often yield limited effects on enzymatic selectivity, multiple-site mutations by combining mutations allow for a more comprehensive modification of the enzyme's active site, efficiently enhancing the specificity (Huang et al., 2023; Sandström et al., 2012). By simultaneously introducing mutations at key residues, researchers can fine-tune the enzyme's interaction with substrate molecules, reducing the hydrolysis of long-chain fatty acids while maintaining or even improving the hydrolysis of short and medium-chain fatty acids (Yang et al., 2002) essential for cheese flavor development.

This study aimed to optimize PCI_Lip for industrial applications by refining its substrate selectivity, and improving its functional properties. With the multiple site mutation approach mutants of PCI_Lip with superior lipolytic characteristics shall be identified. These mutants can help to fill the lack of non-animal derived lipolytic enzymes in dairy industries. Molecular dynamics simulations were employed to systematically analyze its structure-function relationship, providing a foundation for targeted modifications. Based on this information, we designed and generated a mutant library comprising three rounds of mutagenesis. After recombinant expression and subsequent purification, the library was tested on substrate selectivity to identify mutants with altered chain length selectivity. Through multiple-site mutations, we aimed to effectively minimize the hydrolysis of long-chain fatty acids while maintaining activity toward short to medium-chain fatty acids. Kinetic analysis was further performed to confirmed the improved hydrolytic efficiency and specificity of the engineered variants. Finally, we applied these optimized mutants in cheese production. Sensory evaluation combined with SPME-GC–MS analysis were applied to evaluate improved substrate specificity regarding a positive impact on the taste of the cheese, highlighting the potential as superior alternatives to traditional lipases in cheese manufacturing.

## 2 Materials and methods

### 2.1 Cloning

The GenBank file for PCI_Lip can be found under accession number OL364849. For the design of the primers for PCI_Lip without lid Geneious v.9.1.8 software (Biomatters, Auckland, New Zealand) was used to create the primers with overlaps of the particular fragments. Primers were received from Biomers.net GmbH (Ulm, Germany). The respective fragments were amplified in a polymerase chain reaction (PCR). The PCR preparation contained 2.5 μL of each primer, 1 μL dNTPs (Thermo Fisher, Kandel Germany), 10 μL 5× Phusion HF buffer (Thermo Fisher GmbH), 0.5 μL Phusion polymerase (Thermo Fisher) and 0.5 μL of template and was filled up with water to a total volume of 50 μL. To apply different annealing temperatures, aliquots of 10 μL were prepared. The reaction was started by an initial denaturation step at 98 °C for 120 s followed by denaturation at 98 °C for 30 s, annealing at 47–51 °C for 30 s, elongation at 72 °C for 150 s repeated in 35 cycles and a final elongation at 72 °C for 300 s. For all PCRs T100™ thermal cycler (Bio-Rad Laboratories, Munich, Germany) was used. After the reaction the aliquots were pooled, 1 μL of *Dpn*I (Thermo Fisher) was added and incubated at 37 °C for 2 h. The fragment was checked in an agarose gel electrophoresis. Therefore 9 μL of sample were mixed with 1 μL of Midori Green Direct (Biozym Scientific, Hessisch Oldendorf, Germany) and loaded on a 1% agarose gel. Electrophoresis was performed at 100 V and 1000 mA for 1 h. For evaluation the gel was exposed to UV light. After confirmation the fragment was joint using Gibson-Assembly (New England BioLabs, Frankfurt a. M., Germany). 2 μL of PCR products were added to 10 μL of 2× Gibson Assembly Master Mix and filled up with water to a total volume of 20 μL. The preparation was incubated for 15 min at 50 °C. The product of Gibson-Assembly was used for the transformation of chemo competent *Escherichia coli* 10 *β* (New England BioLabs). Overnight cultures in lysogeny broth (LB) medium and kanamycin (Carl Roth, Karlsruhe, Germany) as selection marker were incubated at 37 °C, 180 rpm. After incubation the cells were harvested by centrifugation and used for plasmid isolation using the NucleoSpin® Mini kit for plasmid DNA (Macherey-Nagel, Düren, Germany) according to manufacturer's instructions. Isolated plasmids were sequenced by Eurofins Genomics (Ebersberg, Germany) and sequencing results were analyzed using Geneious 9.1.8 software. Plasmids with positive results were transformed into *E. coli* BL21 (DE3) Gold (New England BioLabs), for recombinant expression.

### 2.2 Protein engineering

Mutants of PCI_Lip were prepared according to Broel et al. The primer design for site-directed mutagenesis was based on the QuikChange™ protocol (Stratagene, La Jolla, CA, USA). For multiple variants the template plasmids contained the respective mutation. For the double mutant L302G + L305A, a single pair of primer incorporating both mutations were used due to close mutation sites. All used primers are shown in Table S1. For the mixture of PCR and PCR reaction conditions see Broel et al.. After PCR reaction the aliquots were pooled and *Dpn*I-digestion was performed by adding 1 μL of DpnI (Thermo Fisher) and incubation at 37 °C for 2 h. Firstly, the digested PCR products were transformed into chemocompetent *E. coli* 10 *β* (New England BioLabs). Positive transformants were selected on LB-kanamycin (Carl Roth) agar plates and used to prepare overnight cultures with adjacent plasmid isolation as described above (Broel et al., 2022). Isolated plasmids were sequenced by Eurofins Genomics (Ebersberg, Germany) and sequencing results were analyzed using Geneious v.9.1.8 software (Biomatters). In a second transformation approved plasmids were transformed into *E. coli* BL21 (DE3) (New England BioLabs) with chaperone plasmid pG-KJE8 (TaKaRa, Kusatsu, Japan) for recombinant expression.

### 2.3 Enzyme production and purification

The cultivation was done according to Broel et al. *E. coli* pellets from 400 mL of culture were resuspended in 12 mL of 50 mM sodium phosphate (Carl Roth) buffer, pH 7.5, 0.3 M NaCl (Carl Roth). Cultivations of mutants were conducted once, and mutants applied in cheese production were cultivated in triplicates. For lysis sonication was applied three times for 2.5 min, 5 cycles and 50% amplitude (sonifier MS72, microtip diameter 5 mm, Bandelin Electronic, Berlin, Germany). The lysate was centrifuged at 14,000 rpm and 4 °C for 15 min. For purification immobilized metal ion affinity chromatography (IMAC) and desalting was performed according to Broel et al. Expression was checked by performing sodium dodecyl sulfate polyacrylamide gel electrophoresis (SDS-PAGE) (Broel et al., 2022; Laemmli, 1970).

For PCI_Lip without lid which was expressed as inclusion bodies application note 18–1134-37 AC by GE Healthcare (Düsseldorf, Germany) for refolding was followed (GE Healthcare Bio-Sciences AB, 2020) (Supplementary material S1.1 Protein refolding). Subsequent desalting and SDS-PAGE were conducted as described above. The purity of all mutants was determined by densitometry (Table S3) using ImageJ v.1.54r.

### 2.4 Determination of enzyme activity

Lipolytic activity of the enzymes was tested in photometric assays using *para*-nitro phenyl (pNP) fatty acid esters of different chain length as model substrates. For PCI_Lip mutants pNP-acetate (pNPA) (Sigma Aldrich), pNP-butyrate (pNPB) (Th. Geyer, Renning, Germany), pNP-valerate (pNPV) (Sigma Aldrich), pNP-hexanoate (pNPH) (TCI Europe, Heuchelheim, Germany), pNP-octanoate (pNPO) (Alfa Aesar, Karlsruhe, Germany) and pNP-palmitate (pNPP) (Sigma Aldrich) were deployed. For the substrates pNPA (Sigma Aldrich), pNPB (Th. Geyer), pNPV (Sigma Aldrich), pNPH (TCI Europe) and pNPO (Alfa Aesar) an esterase assay was conducted at 30 °C with an optical path of 0.64 cm, *ε*_pNP_ = 0.00985 L ∙ μmol^−1^ ∙ cm^−1^ and Triton X-100 as surfactant according to Broel et al. A lipase assay was performed with the substrate pNPP (Sigma Aldrich) at 37 °C with an optical path of 0.8 cm, *ε*_pNP_ = 0.0183 L ∙ μmol^−1^ ∙ cm^−1^ and deoxycholate and gum arabic as surfactants according to Broel et al. In both assays blanks were measured substituting the lipase by potassium phosphate buffer (PPB). One unit of enzyme activity is defined as the amount of enzyme that produces 1 μmol of pNP in one minute in the respective assays. For the calculation of specific activity, protein concentration of the samples was determined according to the method of Bradford (Bradford, 1976; Broel et al., 2022). Briefly, an external calibration with bovine serum albumin (Carl Roth) dissolved in water between 3.125 μg ∙ mL^−1^ and 200 μg ∙ mL^−1^ was prepared. 25 μL of samples and standards were mixed with 200 μL of ROTInanoquant (Carl Roth) in a 96-well microplate. The absorbance was measured at 450 nm and 590 nm. Statistic evaluation was performed by one way ANOVA with a significance level of 0.05 in OriginPro 2023 (OriginLab, Northampton, USA).

### 2.5 Biochemical characterization

For the PCI_Lip mutants S163M + L302G, I245F + L302G, L302G + L305A anL302G + I529D Michaelis-Menten kinetics were determined for the substrates pNPB, pNPH and pNPO. To determine the kinetics different concentrations of the respective substrates were applied in esterase assays conducted as described above. Every measurement was taken in triplicates. The data were subjected to OriginPro 2023 for further analysis. Enzyme activities were plotted against the substrate concentrations using a non-linear fit (category: enzyme kinetics; function: Michaelis-Menten; iteration algorithm: Levenberg-Marquardt; Eq. 1).

(1) $$y=\frac{v_{\max}∙x}{K_{M}+x}$$

### 2.6 Production of feta-type brine cheese using PCI_Lip mutants

To examine the effects of different PCI_Lip mutants on the cheese flavor mutants F91L, S163M, I245F, L302G, L305A, I529D, F91L + I245F, F91L + L305A, S163M + L302G, I245F + L302G, L302G + L305A and L302G + I529D as well as the WT were applied in the production of Feta-type brine cheese. Cheese production was conducted according to Broel et al. Briefly, for each preparation 3 L of milk were used. Milk fat content was 4.2% adjusted with cream. Lactic acid culture (Lyofast M036L), calcium (0.3 mL of 34% *m/v* CaCl_2_) and the lipase were added and incubated at 33 °C for 45 min. For comparison one cheese sample was prepared without the addition of lipolytic enzymes. As a reference the commercially available PGE opti-zym z10uc (0.35 g ∙ (10*L*)^−1^, optiferm, Oy-Mittelberg, Germany) was added to one cheese preparation. One U of each mutant was added to the cheese samples, except for mutants L302G + L305A only 0.5 U were added due to low amount of purified enzyme for this mutant. The amount of lipase used was based on the activity of the respective mutant to pNPO in the esterase assays which corresponds to 2.0 to 9.5 mL of purified enzyme. Then, 0.75 mL microbial rennet (opti-ren micro, 220 IMCU; optiferm,) was added. The mixture was set to rest for 1 h. After formation of cheese curd, it was cut into cubes and transferred to molds, where the whey could drain. After resting for 24 h, the loafs were submerged brine (20% *m/v* NaCl, pH 5.2) for 50 min. After 30 days of ripening at 13 °C the edges of the samples were removed to get rid of the preservative natamycin. The samples were subjected to analysis of volatile free fatty acids (vFFA) by solid phase micro extraction gas chromatography–mass spectrometry (SPME-GC–MS).

### 2.7 Sensory evaluation and vFFA analysis of feta-type brine cheese samples

After 30 days of ripening the cheeses prepared with different mutants were used for analysis. All cheese samples were prepared in cubes of 1 × 1 × 1 cm^3^ and labeled with a random three-digit code. The panel consisted of four female and four male people, two of which were smokers. The age of the panel reached from 24 to 44 years of age. Evaluation was based on defined attributes (smell: piquant, sour, goat-like, soapy, dairy, intensity; while for the taste: piquant, sour, goat-like, soapy, dairy, bitter, intensity are used), which were rated on a scale from 0 to 5 with increasing perception. Meanwhile, the profile of vFFAs in cheese samples were analyzed by SPME-GC–MS (Supplementary material S1.2, Broel et al., 2022.). Statistic evaluation was performed by one way ANOVA with a significance level of 0.05 in OriginPro 2023.

### 2.8 Molecular dynamics (MD) simulation

The structure of PCI_Lip was generated by Alphafold3. The enzymatic reaction system was constructed using Packmol (Martínez et al., 2009), and molecular dynamics (MD) simulations were performed using NAMD (Phillips et al., 2020). Details of the MD simulation protocol follow those described in our previous work (Zhang et al., 2024). The lipase protein conformation was centrally positioned within a cubic water box measuring 90 × 90 × 90 Å^3^, with 10 triglyceride molecules incorporated into each system. Four distinct model triglycerides, differing in fatty acid chain length (C4:0, C10:0, C16:0, C18:0, and C18:1) were used, leading to the construction of four separate simulation units. To optimize the system and eliminate unfavorable atomic contacts, energy minimization was first conducted using a 5000-step conjugate gradient method. Subsequently, MD simulations were initiated, gradually increasing the temperature from 0 K to 303 K under NPT conditions over 200 ps. Three independent simulations were executed for each system under identical conditions, each lasting 200 ns, with a time step of 2 fs to generate the PCI_Lip lipase trajectory files. For all MD processes, energy minimization was performed using the conjugate gradient method, while system equilibration was achieved *via* Langevin dynamics. Finally, the simulation data were analyzed using VMD software (Nakkala et al., 2022).

### 2.9 Molecular docking

Molecular docking was conducted using AutoDock Vina v.1.2.3, developed by Arthur J. Olson at the Scripps Research Institute (La Jolla, CA, USA), employing the Broyden-Fletcher-Goldfarb-Shanno optimization algorithm. Ligand and receptor preparation was carried out using the AutoDock Tools package. A semi-flexible docking approach was utilized, with the active site residue S213 designated as the docking center. All other docking parameters remained at their default settings. Four model triglycerides with distinct fatty acid chain lengths (C4:0, C10:0, C16:0, C18:0, and C18:1) were used in the simulations.

## 3 Results and discussion

### 3.1 Analysis of the active site

**Fig. 1:**
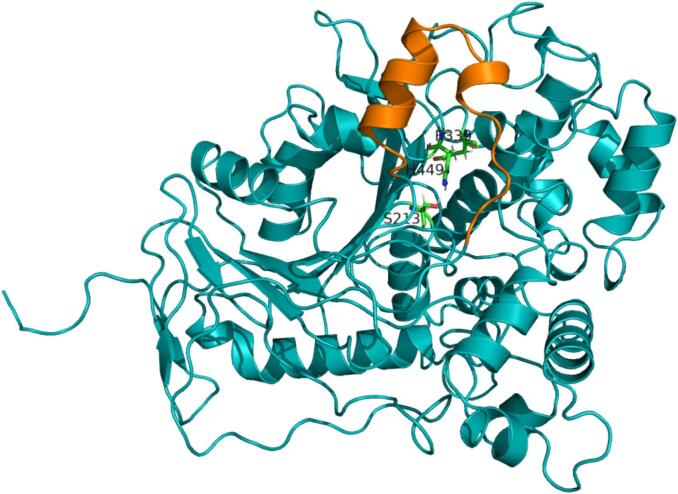
AlphaFold 3 model of PCI_Lip. The lid region (A69–F91) is marked in orange. The amino acid residues of the catalytic triad (S213, H449, E339) are shown in green and labeled with the respective one letter code. (For interpretation of the references to colour in this figure legend, the reader is referred to the web version of this article.)

**Fig. 2:**
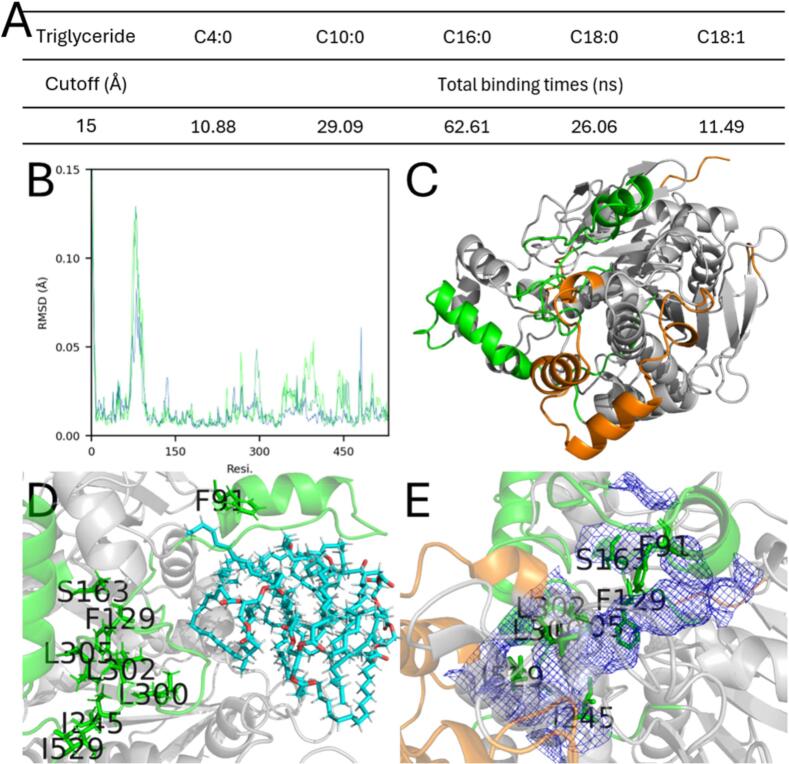
Molecular docking and simulation analysis of the PCI_Lip. (A) The total binding time of the substrates entering the active center, calculated as the sum of three parallel simulations. It represents the total duration during the three carbonyl carbon atoms of the triglyceride enter the active center. (B) RMSD of each residue when C16:0 was used. (C) 3D structure of PCI_Lip. Orange: flexible region distant from the active pocket. Green: flexible region forming part of the binding pocket. (D) Snapshot of C16:0 binding to the active pocket. The frame was selected at the point of minimum distance. C16:0 was shown as cyan sticks. (E) The binding pocket of PCI_Lip shown as blue mesh. (For interpretation of the references to colour in this figure legend, the reader is referred to the web version of this article.)

**Fig. 3:**
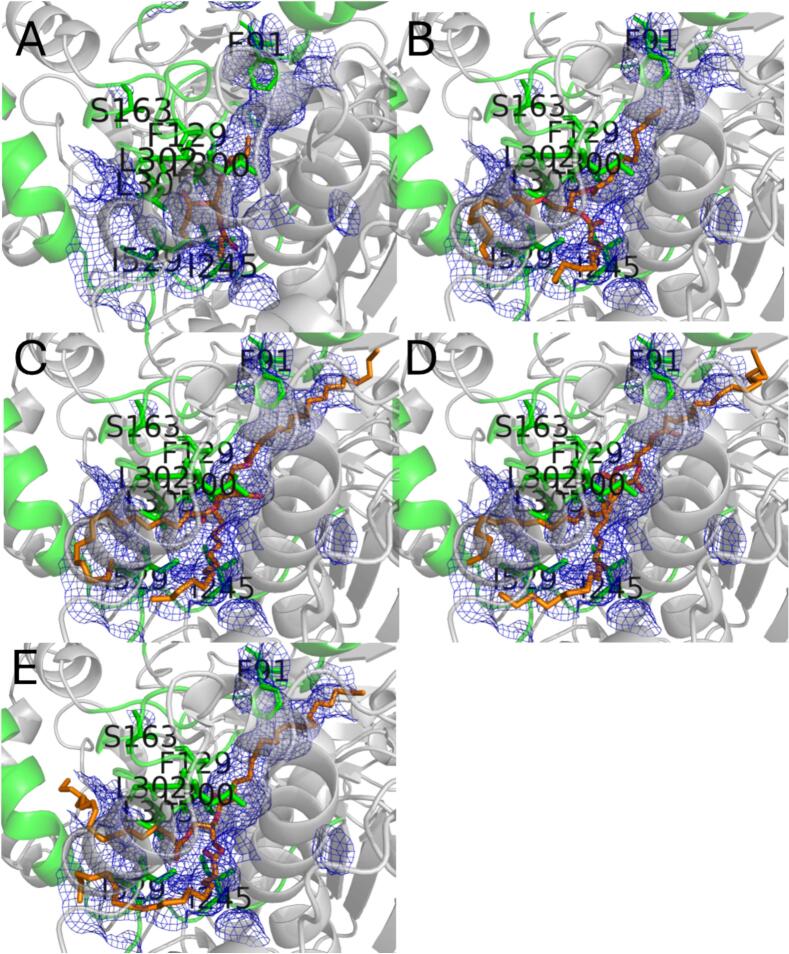
Docking of C10:0 (A), C16:0 (B), C18:0 (C), and C18:1 (D) in the binding pocket of PCI_Lip (blue mesh). Substrates were shown as orange sticks. Green: flexible region forming part of the binding pocket. (For interpretation of the references to colour in this figure legend, the reader is referred to the web version of this article.)

PCI_Lip displays a typical interfacial-activated lipase with a highly conserved catalytic center (S213, H449, E339; Fig. 1). To modify its hydrolytic selectivity, it was essential to identify the effective triglyceride-binding pocket. Therefore, MD simulations were used to investigate the hydrolytic selectivity of PCI_Lip toward four triglycerides with different carbon chain lengths, which were the main fatty acids in milk (Fig. S1) (Broel et al., 2022; Wang et al., 2025). As shown in Fig. 2A, the WT PCI_Lip exhibited the longest binding time for C16:0 indicating a higher selectivity for this fatty acid. During triglyceride binding, the crucial lid and binding pocket must undergo structural changes to accommodate the sterically hindered triglyceride molecules entering the active pocket. Thus, the RMSD of the carbon backbone was calculated for each residue of PCI_Lip during the simulation of the four substrates (Figs. 2B and S2). The flexible region was highlighted in Fig. 2C, with special attention paid to the green flexible region as it is orientated in close proximity to the active center. The most flexible region was the interfacial-activated lid, which covers the residues A69–F91. Fig. 2D illustrates the frame in which the distance between C16:0 and the active site S213 (not shown) was at its minimum. In contrast, the other three triglyceride substrates failed to fully enter the active pocket. This may be attributed to the higher steric hindrance of C18:0 and C18:1, as well as interference caused by multiple substrate molecules present simultaneously in the reaction system. For C4:0, instability might result from its small size and high flexibility, making it difficult to position them in a potential active conformation within the pocket. Meanwhile, the smaller C10:0 substrate's failure to enter effectively could be due to relatively low affinity for the PCI_Lip's for this substrate. The MD simulation revealed that, in addition to the lid containing F91, significant structural changes occurred in the loops or *α*-helices where F129, S163, I245, L300, L302, L305, and I529 were located, allowing adaptation to triglyceride entry into the active center. As shown in Fig. 2E, the initial conformation of PCI_Lip formed a complete tunnel-shaped binding pocket by the residues F91, F129, S163, I245, L300, L302, L305, and I529. This tunnel shape could lead to an easy penetration of the esterified fatty acid chains of the triglycerides. Considering the substantial steric hindrance of triglyceride molecules, as well as solvation effects and intermolecular interactions, it was nearly impossible for the total substrates to perfectly enter the active center solely through MD simulations. To further validate this finding, molecular docking using S213 as the center all four triglyceride molecules were performed (Fig. 3). The results demonstrated that all four triglycerides could fit perfectly into the binding pocket (blue mesh), further supporting the above-mentioned hypothesis.

**Fig. 4:**
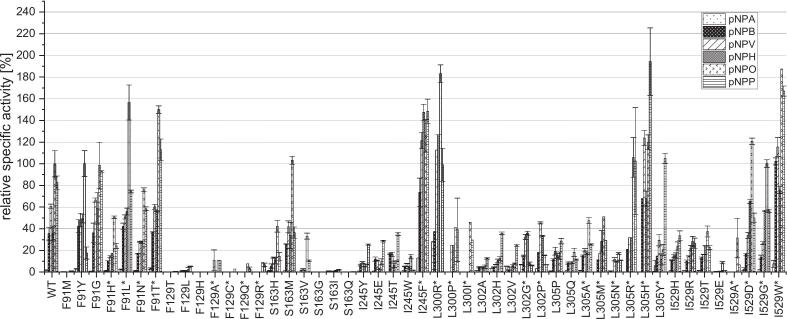
Relative specific activity [%] of PCI_Lip single mutants against six *para*-nitrophenyl (pNP) fatty acid esters of different chain length as model substrates. All specific activities are compared to the specific activity of the WT against pNPO, which was set to 100%. All photometric assays were performed in triplicates. Standard deviation of the measurements is described by the error bars. Mutants marked with * have been characterized by Broel et al. For statistical evaluation see Fig. S19.

To underline the importance of the residues valuable data on mutations in the binding pocket of PCI_Lip, as shown in Fig. 4 and S7, were provided by Broel et al., which produced and partially characterized 26 variants at seven of the eight relevant substrate binding amino acids in the tunnel. Among these mutants, F91L stood out, displaying a hydrolysis profile similar to that of the WT. The activity of this mutant toward pNPA, pNPB, pNPV, pNPH, and pNPO remained nearly unchanged, whereas its activity against pNPP was reduced by more than 50%. This reduction in activity might be attributed to the location of F91 at the entrance of the binding tunnel. When F91 was mutated to a smaller amino acid leucine (L), it might be more flexible and rotate to block the larger substrate pNPP to access the active center, thereby reducing the hydrolysis. Furthermore, when mutations performed at I245, L300, L302, L305, and I529, significant changes in the enzyme's catalytic performance (activity and selectivity) were observed, indicating the sensitivity of these sites. This suggests that these residues play a direct role in substrate binding, thereby directly influencing hydrolytic selectivity. For example, when L302 was mutated to either glycine (with lower steric hindrance) or proline (with greater rigidity), the hydrolysis profile underwent a significant change. Although the overall activity decreased, the selectivity for medium-chain fatty acids was markedly enhanced. Although in the study by Broel et al., mutants at position F129 either exhibited almost complete loss of activity or failed to express. Additionally, since S163 is located at a highly critical position (Figs. 2E and 3), it was still included as a potential mutation site. Therefore, our selected potential mutation sites are F91, F129, S163, I245, L300, L302, L305, and I529.

### 3.2 Comparison of the PCI_Lip with another mushroom-derived lipase without lid

**Fig. 5:**
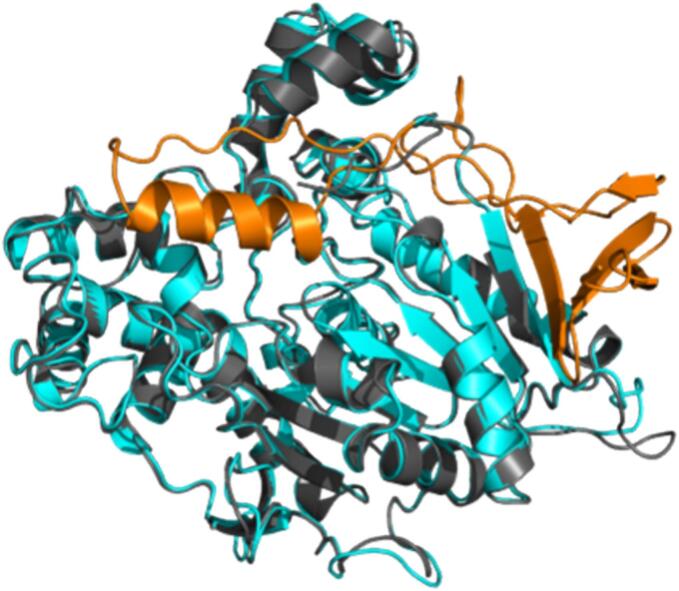
Structure alignment PCI_Lip (cyan; lid orange) and PCE_Lip (black). The additional structure in PCI_Lip compared to PCE_Lip is highlighted in orange, which includes the crucial lid. (For interpretation of the references to colour in this figure legend, the reader is referred to the web version of this article.)

Another lipase derived from the Basidiomycota *Phlebia centrifuga* (PCE_Lip) has been identified using Enzyme Miner (Hon et al., 2020) searching for similar lipases to PCI_Lip. With a putatively improved solubility index of 0.6771 and a sequence similarity of 66.5% as this enzyme was 2022 the only entry with high similarity (>65%) with improved solubility index compared to the PCI_Lip with an index of 0.451. The alignment of AlphaFold3 models of both PCI_Lip and PCE_Lip revealed a high structural similarity of the lipases. The RMSD was only 0.444 Å basing on their whole structures. Considering the predicted superior solubility of PCE_Lip, the recombinant expression was attempted with *E. coli* BL21 (DE3) Gold and *E. coli* BL21 (DE3) with five different chaperones provided by TaKaRa Bio Inc. No soluble expression of PCE_Lip was found in any of the different attempts. PCE_Lip could only be expressed as insoluble inclusion bodies (Fig. S10). The inclusion bodies were subjected to an on-column refolding to get soluble enzyme for determining the activity. The activity was measured photometrically using pNP fatty acid esters as model substrates. The specific activity of PCE_Lip against any pNP fatty acid ester was close to the detection limit, which is an indication that the catalytic behavior of the PCE_Lip is different to the PCI_Lip. Comparing the two lipase models the most significant difference is the *N*-terminal region of PCI_Lip (Fig. 5)The region M1 to V90 in PCI_Lip is assumed to be the putative lid area and not present in PCE_Lip (Fig. S8). Lids are mobile domains exposing the substrate channel when there is contact to the non-polar substrate. This interfacial activation is often seen as another criterion for the classification as a lipase. However, lipases can also have immobile elements covering the substrate channel and restricting access to the substrates, which are referred to as caps (Bauer et al., 2020). As the PCE_Lip does not possess a lid, it can still be categorized as a lipase as a lid is no mandatory structural element. The resolution of the crystal structure of lipase A from *Bacillus subtilis* is another example for a microbial lipase without lid domain. This lipase is showing a minimal *α*/*β*-hydrolase fold with compact globular structure lacking a lid or cap domain (Van Pouderoyen, Eggert, Jaeger and Dijkstra, 2001).

Figs. S3–S7 illustrate the kinetic behaviors of five triglycerides interacting with PCI_Lip. Despite variations in effective diffusion trajectories, a common feature among all cases was the necessity for triglycerides to pass through the critical lid region (A69–F91) before reaching the active site for hydrolysis (Figs. S3A–S7A). Structural alignment analysis revealed that most of the protein maintains its rigidity due to stable secondary structures, the lid region undergoes significant conformational changes due to its flexible loops and small *α*-helix (Figs. S3B–S7B). These structural dynamics play a key role in regulating substrate access, while each substrate exhibited distinct binding behaviors. MD simulations revealed complex and individual movements of the lid domain depending on the different substrates. The individual changes of the lid domain are affected by the different diffusional channels of different substrates (Fig. S3C + D–7C + D). C10:0 showed notable trajectory aggregation at the back side of the active pocket, though it could not directly pass the *α*-helix structures due to steric hindrance. Instead, it accessed the active site through adsorption-desorption events or lateral movement along the protein surface, ultimately crossing the lid (Fig. S4C). C16:0 displayed a higher trajectory density on the protein surface and a larger opening angle, facilitating its passage through the lid. Residue-specific affinity analysis identified the lid as the primary binding site for C16:0 (Fig. S5D), making it the most effective substrate for activating the lid. For C18:0, an additional aggregation site appeared around another *α*-helix and a *β*-strand (Fig. S6D), increasing overall hydrophobic interactions but reducing regional selectivity. Its smaller opening angle and steric resistance limited entry into the active site, decreasing binding efficiencies. Meanwhile, C18:1 exhibited random trajectory scattering across the reaction microunit (Fig. S7A), with minimal aggregation in the active pocket, suggesting weak and unstable binding. Despite lid activation, the opening angle remained relatively small, further hindering stable substrate binding. As a lid domain can strongly impact the overall characteristics of a lipase in this study it was decided to add the putative lid domain of PCI_Lip to the structurally similar PCE_Lip (Fig. 5).

To add the lid of PCI_Lip to PCE_Lip the nucleotides coding for amino acids M1 to L85 in PCI_Lip were integrated into the pET28a(+) carrying the PCE_Lip gene. Recombinant expression in *E. coli* BL21 (DE3) with the chaperones DnaK-DnaJ-GrpE and GroES-GroEL led to the formation of inclusion bodies. To get soluble enzyme for further analysis an on-column refolding was performed. The activity directly after the refolding was comparable to the one of PCE_Lip without a lid, providing insides that the addition of a lid barely improved the activity. This gives reason to consider other substrate as native to this enzyme that may be less lipophilic as the enzyme does not require a lid domain.

To further investigate the role of the putative lid structure in PCI_Lip another mutant was created coding for PCI_Lip without lid domain (Fig. 5). Recombinant expression using the same expression host as before also led to the formation of insoluble inclusion bodies and on-column refolding has been conducted to investigate the influence of the lid on the activity of this lipase. In comparison to the WT the activity is drastically reduced by more than 100-times. The drastic changes in the activity and solubility indicated that the putative lid domain in PCI_Lip is an essential structural part of this enzyme.

### 3.3 Tailoring the PCI_Lip *via* several rounds of mutagenesis

For mutations the positions F91 in the putative lid domain, S163 near the substrate channel and positions F129, I245F, L302, L305 and I529 in the substrate channel were mutated *in silico* with the 19 other amino acids (Broel et al., 2022). In total 19 out of 21 predicted plasmids containing mutations could be constructed, and the mutants could be expressed and purified (Fig. S9). Overall, most of those mutants exhibited quite low activity, in the range of 1 to 40% (Fig. 4). Mutant F91Y is standing out of this set as it displays a similar hydrolysis profile as the WT. Comparing the hydrolysis profiles of the three F91 mutants in these experiments to the four single mutants from Broel et al. the exchanges led mainly to mutants with similar profiles as the WT but with reduced activities. The position is located in a flexible domain of the putative lid area (Fig. 3). Furthermore, all mutants at positions F129 and S163 have lost almost all activity. F129 seems to be a crucial position for the enzyme activity as the single mutants predicted to have a positive impact also barely show activity. All mutations at I245, L302, L305 and I529 lead to low but still active mutants exhibiting high activities toward pNPP. The I245 mutants all display the highest affinity toward pNPP with similar hydrolysis profiles. Mutating L302 to G or P to generate improved mutants preferentially hydrolyzing pNPV and pNPH. Exchanging L for A or V reduced the activity to pNPV and pNPH but enhanced affinity for long chain fatty acids. All involved amino acids carry hydrophobic residues only differing in steric matters. Interestingly, small residues seem to support the hydrolysis of short chain substrates, while bigger residues promote hydrolysis of long chain substrates. Mutating L302 to a polar H led to a higher affinity toward pNPP. The exchange to a polar residue might hinder the short chain substrates in fully entering the substrate channel. The effects of different amino acid residues are quantitatively reflected by the selectivity ratios (Table 1). In case of position L302, a change to a smaller residue such as G markedly increased the pNPH/pNPP ratio whereas substitutions to moderate bulky residues give ratios similar to the WT. Similar effects appear at position L305. Substitutions to smaller residues increase the selectivity ratio while larger and polar residues decrease it. At the same time mutation may support the slightly different binding mode described for pNPP (Broel et al., 2022). Overall, the photometric characterization of mutants with impaired characteristics is in accordance with the predictions from MD simulations underlining that the model is appropriate.

**Table 1:** List of single, double and triple mutants in the mutant library of PCI_Lip. Mutants marked with * have been characterized by Broel et al., 2022. For the overview the positions of the respective mutations inside the protein structure are listed. The ratios of pNPO/pNPP and pNPH/pNPP are calculated from the specific activities of the mutants against pNPO or pNPH in relation to the specific activities against pNPP, respectively (n.a. – not applicable).

Mutant	Position of mutation	Ratio pNPO/pNPP	Ratio pNPH/pNPP	Protein concentration [μg mL^−1^]	
WT	–	1.2	0.4	511.7 ± 23.4	
F91M	lid	n.a.	59.8	315.8 ± 12.7	
F91Y	lid	5.7	n.a.	313.7 ± 5.4	
F91G	lid	1.1	0.6	1075.0 ± 12.5	
F91H*	lid	2.1	0.7	303.9 ± 19.2	
F91L*	lid	2.1	1.2	244.4 ± 49.5	
F91N*	lid	1.3	0.5	338.9 ± 16.3	
F91T*	lid	1.3	0.5	212.2 ± 4.4	
F129T	substrate channel	0.1	67.4	426.8 ± 17.3	
F129L	substrate channel	0.4	n.a	313.8 ± 14.7	
F129H	substrate channel	n.a.	3.7	439.1 ± 11.8	
F129A*	substrate channel	1.0	n.a	76.2 ± 2.4	
F129C*	substrate channel	n.a	n.a	94.2 ± 16.9	
F129Q*	substrate channel	2.3	n.a	108.4 ± 2.6	
F129R*	substrate channel	1.3	n.a	93.9 ± 2.3	
S163H	near substrate channel	2.9	0.7	266.5 ± 14.2	
S163M	near substrate channel	2.8	0.9	289.8 ± 41.8	
S163V	near substrate channel	3.2	0.2	231.4 ± 12.0	
S163G	near substrate channel	0.4	0.6	895.7 ± 14.7	
S163I	near substrate channel	0.4	0.4	197.3 ± 3.4	
S163Q	near substrate channel	n.a.	0.2	421.9 ± 13.3	
I245Y	substrate channel	0.3	0.2	553.9 ± 7.6	
I245E	substrate channel	0.3	0.1	376.0 ± 16.9	
I245T	substrate channel	0.3	0.2	417.9 ± 18.9	
I245W	substrate channel	8.2	2.5	526.7 ± 72.9	
I245F*	substrate channel	0.8	1.0	96.4 ± 4.8	
L300R*	substrate channel	1.8	1.3	89.0 ± 1.3	
L300P*	substrate channel	1.1	0.6	50.8 ± 4.8	
L300I*	substrate channel	1.5	n.a.	18.2 ± 3.8	
L302A	substrate channel	0.4	0.3	518.4 ± 22.7	
L302H	substrate channel	0.3	0.3	316.4 ± 13.3	
L302V	substrate channel	0.3	0.2	498.5 ± 3.2	
L302G*	substrate channel	2.3	10.0	170.8 ± 11.9	
L302P*	substrate channel	2.3	4.9	173.0 ± 10.7	
L305P	substrate channel	0.5	0.5	344.6 ± 49.7	
L305Q	substrate channel	1.6	0.8	344.6 ± 49.7	
L305A*	substrate channel	1.9	1.4	170.1 ± 3.0	
L305M*	substrate channel	1.7	1.0	73.6 ± 1.5	
L305N*	substrate channel	1.6	0.9	135.7 ± 4.5	
L305R*	substrate channel	1.0	0.3	39.5 ± 0.4	
L305H*	substrate channel	0.6	0.3	104.7 ± 3.4	
L305Y*	substrate channel	0.2	0.1	142.5 ± 1.9	
I529H	substrate channel	0.7	0.5	516.7 ± 11.4	
I529R	substrate channel	1.1	0.8	532.9 ± 22.9	
I529T	substrate channel	1.7	1.1	511.7 ± 23.4	
I529E	substrate channel	5.7	0.5	916.5 ± 176.4	
I529A*	substrate channel	4.6	n.a.	39.8 ± 2.0	
I529D*	substrate channel	2.4	1.3	182.1 ± 26.5	
I529G*	substrate channel	1.8	1.0	240.7 ± 30.2	
I529W*	substrate channel	1.1	0.5	112.2 ± 1.1	
F91L + S163M	lid - near substrate channel	1.2	0.5	155.4 ± 19.7	
F91L + I245F	lid - substrate channel	4.0	2.2	238.3 ± 1.3	
F91L + L302G	lid - substrate channel	9.2	25.7	125.3 ± 4.9	
F91L + L305A	lid - substrate channel	3.4	1.7	517.3 ± 11.0	
F91L + I529D	lid - substrate channel	4.0	2.5	261.8 ± 57.8	
S163M + I245F	near substrate channel - substrate channel	0.4	0.4	221.5 ± 41.7	
S163M + L302G	near substrate channel - substrate channel	2.6	11.6	142.4 ± 26.3	
S163M + L305A	near substrate channel - substrate channel	0.6	0.3	201.2 ± 26.9	
S163M + I529D	near substrate channel - substrate channel	0.7	0.5	204.7 ± 22.6	
I245F + L302G	substrate channel - substrate channel	1.3	16.8	292.3 ± 6.3	
I245F + L305A	substrate channel - substrate channel	1.8	4.6	240.7 ± 12.1	
I245F + I529D	substrate channel - substrate channel	1.7	0.3	255.3 ± 8.8	
L302G + L305A	substrate channel - substrate channel	1.0	10.4	168.2 ± 32.7	
L302G + I529D	substrate channel - substrate channel	2.7	2.2	255.2 ± 9.6	
L305A + I529D	substrate channel - substrate channel	2.5	1.2	277.9 ± 10.9	
F91L + I245F + L302G	lid – substrate channel - substrate channel	1.3	8.0	378.2 ± 15.7	
F91L + I245F + L305A	lid – substrate channel - substrate channel		2.0	2.3	218.1 ± 22.1
F91L + L302G + L305A	lid – substrate channel - substrate channel		0.5	3.0	160.8 ± 28.9
F91L + L302G + I529D	lid – substrate channel - substrate channel		1.4	5.2	219.3 ± 17.3
F91L + L305A + I529D	lid – substrate channel - substrate channel		2.1	1.0	189.7 ± 14.6
I245F + L302G + L305A	substrate channel - substrate channel - substrate channel		0.7	6.3	219.5 ± 15.9
L302G + L305A + I529D	substrate channel - substrate channel - substrate channel		0.4	0.5	179.8 ± 13.0

**Fig. 6:**
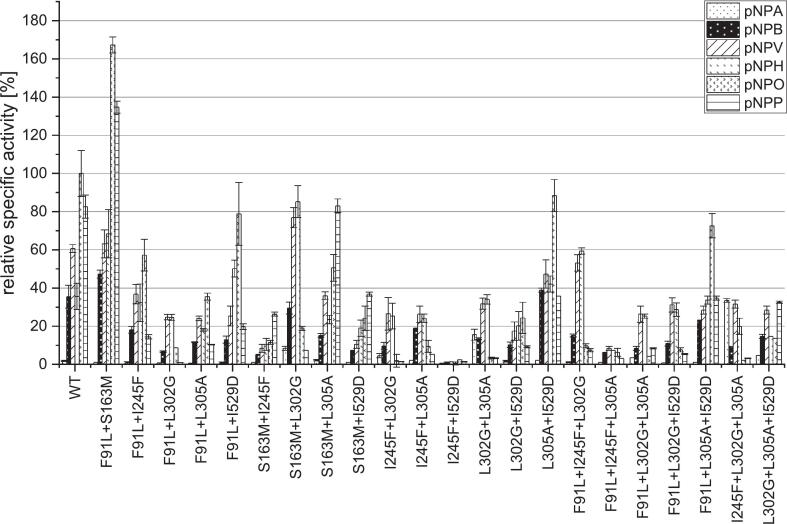
Relative specific activity [%] of PCI_Lip 15 double and nine triple mutants against six *para*-nitrophenyl (pNP) fatty acid esters of different chain length as model substrates. All specific activities are compared to the specific activity of the WT against pNPO, which was set to 100%. All photometric assays were performed in triplicates. Standard deviation of the measurements is described by the error bars. For statistical evaluation see Fig. S20.

In total, 15 gene constructs for double mutants have been created (Table 1 and Table S1) by combining promising single mutations. All 15 double mutants were successfully expressed and purified (Figs. S12 and S13). The hydrolysis profiles of the double mutants are shown in Fig. 6. Except for F91L + S163M all double mutants show lower relative specific activities than the WT. The desired decrease in the hydrolysis to yield palmitic acid is observed in ten double mutants which show better pNPH/pNPP or pNPO/pNPP ratios than the WT (Table 1). For all these double mutants either the ratios pNPO/pNPP or pNPH/pNPP could be improved. In most cases both ratios improved compared to the WT. In the WT enzyme the highest activity is against pNPO. The double mutants F91L + L302G, S163M + L302G, I245F + L302G, I245F + L305A and L302G + L305A primarily show highest activity to pNPH and activity to pNPO is drastically restricted. The activity toward pNPH of I245F + L305A is four times higher than in the WT. The four other mutants mentioned stand out with some of the highest ratios pNPH/pNPP between 10 and 25 within all the examined mutants. Considering both ratios, double mutant F91L + L302G strikes by the highest ratio of pNPO/pNPP of 9.2 being the highest within the whole library as well as a ratio pNPH/pNPP of 25.7 being third highest within the library. The same change of affinity has been observed for the single mutant L302G, also seen in a ten-fold higher affinity against pNPH than pNPP as the WT. These results underline the assumption that the position L302 is crucial for PCI_Lip chain length specificity (Broel et al., 2022). The overall increase of hydrolytic activity elicited by the mutation I245F could not be transferred to its double mutants as these generally exhibit rather low overall activity. After two rounds of mutagenesis three mutants still exhibited a hydrolysis profile similar to the WT but with lower affinity toward pNP palmitic acid. The reduced affinity against pNPP while simultaneously keeping a comparable hydrolysis profile as the WT is resembled in improved ratios of pNPO/pNPP and pNPH/pNPP. These results go along with the hydrolysis profile of the single variant L305A and emphasize the importance of the position L305 for chain length specificity notably affecting its affinity to palmitic acid. The *in silico* analysis revealed that the ester bond of substrates with a long acyl chains is situated more toward the solvent accessible part of the substrate channel (Broel et al., 2022). The mutation L305A seems to emphasize this situation making access for long chain fatty acids more difficult. For cheese making applications the hydrolysis profile ideally should shift to primarily hydrolyzing butyrate, hexanoate and octanoate while minimizing palmitate hydrolysis (Sowa et al., 2022). The hydrolysis profiles of F91L + L302G, S163M + L302G, I245F + L302G, I245F + L305A and L302G + L305A comply with these requirements very well. Additionally, the comparison of both ratios shows that double mutants that are considered best as substitutes have considerably higher values for the pNPH/pNPP than for the pNPO/pNPP ratio. Most of these double mutants display a relative specific activity of 20% to 40% compared to the pNPO activity of the WT, which is comparable to the activity of the single mutant L302G with a similar hydrolysis profile. Combining L302G with the exchange S163M kept the desirable hydrolysis profile, while simultaneously boosting activity to over 80% compared to the WT for pNPH. Combining favorable single mutants did not necessarily show additive or synergistic effects in the second round of mutagenesis. However, the comparison of selectivity ratios of single and double mutants underlined non-linear coupling effects rather than single-site effects. As the examined residues shape the substrate pocket of PCI_Lip we propose cooperative mechanisms of gating effects caused by F91L in the lid domain and a remodeling of the channel geometry by the other mutations leading to restricted access for long chain substrates, while maintaining activity against short to medium chain substrates. Rokyta et al. describe similar observation in microbial and viral systems. Combining beneficial single mutants can result in a mutant that shows less beneficial properties than the single mutants. Even though the single mutants show beneficial properties double mutants can be a step away from the optimum in the fitness landscape (Rokyta et al., 2011).

The most interesting double mutants according to their hydrolysis profiles as well as the pNPH/pNPP and pNPO/pNPP ratios S163M + L302G, I245F + L302G, L302G + L305A and L302G + I529D have been further biochemically characterized by means of Michaelis-Menten kinetics. Therefore, the substrates pNPB, pNPH and pNPO have been chosen as these fatty acids are important for the flavor of piquant cheese varieties. The values obtained for maximal velocity (*v*_max_), Michaelis-Menten constant (*K*_M_), turnover number (*k*_cat_) and catalytic efficiency (*k*_cat_/*K*_M_) are listed in Table 2. The differences of these parameters within the four mutants showcase the changes in hydrolytic activities caused by the amino acid exchanges (Figs. S15–S18). The maximal velocity is highest for pNPH for all the double mutants. Michaelis-Menten constants increase with decreasing chain length of the substrates. Turn over numbers and catalytic efficiencies go along with the hydrolysis profiles obtained in the photometric assays (Fig. 6). Both values are low for pNPO in the analysis of double mutants I245F + L302G and L302G + L305A which displayed very low activity against this substrate in their hydrolysis profiles. In comparison to the catalytic efficiencies of the WT (Broel et al., 2022) the ones of the double mutants are lower for the three substrates. This correlates with the overall lower hydrolytic activity of the double mutants compared to the WT. All catalytic efficiencies and especially the turnover numbers confirm that we were able to change the specificity of PCI_Lip within this second round of mutagenesis rather toward short chain fatty acids. The second round of mutagenesis offered some mutants that exhibited altered chain length specificity with distinguishable changes in pNPH/pNPP and pNPO/pNPP ratios. Additionally, the importance of the positions L302 and L305 for the enzymes chain length specificity have been underlined.

**Table 2:** Kinetic data of PCI_Lip double mutants S163M + L302G, I245F + L302G, L302G + L305A and L302G + I529D for the substrates pNPB, pNPH and pNPO with confidence intervals (95% CI) and determination coefficient (*R*^2^).

	Protein concentration [μmol ∙ L^−1^]	Substrate	*v*_max_ [μmol ∙ min^−1^ ∙ L^−1^]	lower CI *v*_max_ [μmol ∙ min^−1^ ∙ L^−1^]	upper CI *v*_max_ [μmol ∙ min^−1^ ∙ L^−1^]	*K*_M_ [mmol ∙ L^−1^]	lower CI *K*_M_ [mmol ∙ L^−1^]	upper CI *K*_M_ [mmol ∙ L^−1^]	*k*_cat_ [s^−1^]	*k*_cat_/*K*_M_ [s^−1^ ∙ mol^−1^ ∙ L]	*R*^2^
S163M + L302G	2.20 ± 0.21	pNPB	40.83	25.51	56.16	1.31	0.45	2.18	0.309	236	0.95
		pNPH	91.88	74.40	109.35	0.65	0.40	0.89	0.696	1071	0.95
		pNPO	13.46	9.10	17.81	0.07	−0.04	0.18	0.102	1456	0.50
I245F + L302G	2.12 ± 0.06	pNPB	42.07	25.69	58.44	1.81	0.70	2.94	0.331	183	0.97
		pNPH	95.43	74.11	116.75	0.75	0.41	1.10	0.750	1000	0.97
		pNPO	11.18	8.42	13.47	0.15	0.05	0.25	0.088	586	0.81
L302G + L305A	1.46 ± 0.03	pNPB	23.00	13.94	32.06	1.75	0.65	2.86	0.262	150	0.96
		pNPH	30.36	24.44	36.27	0.42	0.19	0.65	0.346	823	0.96
		pNPO	6.53	5.16	7.90	0.13	0.02	0.23	0.074	572	0.84
L302G + I529D	2.35 ± 0.10	pNPB	26.50	18.85	34.14	1.41	0.67	2.16	0.188	133	0.96
		pNPH	50.45	44.17	56.74	0.50	0.36	0.63	0.358	715	0.96
		pNPO	45.15	39.26	51.05	0.39	0.29	0.50	0.320	821	0.97

Taking the protein engineering of PCI_Lip a step further a third round of mutagenesis has been accomplished. Here the mutation sides F91L, I245F, L302G, L305A and I529D have been considered. Seven triple mutants have also been successfully expressed and purified (Fig. S14). The relative specific activity of all triple mutants is lower than the WT activity (Fig. 6). The highest activity is shown by F91L + L305A + I529D with over 70% against the substrate pNPO. Secondly mutant F91L + I245F + L302G displays more than 60% of activity in comparison to the WT against pNPH. The other five mutants show rather low activities from 30% and lower. The library of mutants in this study underlines the discrepancy of better fitness due to diversity but simultaneously loss of activity (Ostermeier, 2003). Moreover, the hydrolysis profiles of the triple mutants are more deviate from the profile of the WT as mutants from the first two rounds. . Here, the preferred affinity is predominantly shifted from pNPO to pNPH. Therefore, the significance of position L302 falls into place as five out of seven mutants contain L302G. These mutants resemble the hydrolysis profile of the single mutant. This underlines the postulation by Broel et al. for this side to be crucial for chain length specificity. Interestingly, F91L + I245F + L302G shows a synergistic effect in terms of its hydrolysis profile. The distribution of relative specific activities is mainly influenced by L302G and very similar to the profile of double mutant F91L + L302G. The combination with I245F, which was found to increase the overall activity led to an increase to nearly 60% relative specific activity indicating an additive effect of these mutations (Broel et al., 2022). Especially the third round of mutagenesis demonstrates that small steps in the fitness landscape of a protein can result in different effects as additivity as well as positive or negative coupling. Additivity is seen in F91L + I245F + L302G with its hydrolysis profile being the sum of the individual changes the double and single variants displayed (Lunzer et al., 2005). Positive coupling effects can be observed in all triple mutants except F91L + L305A + I529D and L302G + L305A + I529D as combinations led to the desired reduction in activity to pNPP. But also negative coupling effects are observed for example in the considerable decline in the overall activity particularly noticeable for F91L + I245F + L305A. Coupling is caused by altered interactions of the amino acid residues and can cause beneficial such as stabilizing effects as well as negative effects for example due to van der Waals conflicts (Voigt et al., 2000). The third round of mutagenesis revealed mutants with hydrolysis profiles shifted to mainly hydrolyze pNPH as a result of additive effects of individual mutations. However, overall activity was considerably lower than in the previous rounds, which means the combination of more active variants did not lead to improved variants indicating a valley in the fitness landscape. Hence the third round of mutagenesis is a good example for better fitness of the enzyme due to increased diversity but simultaneously substantial loss of activity (Ostermeier, 2003).

### 3.4 Evaluation of vFFA and sensory of feta-type brine cheese samples

Mutants have been deemed to have a positive impact, when the hydrolysis profile of the WT was shifted to an enhanced hydrolysis of short to medium chain fatty acids. Also, a lowered affinity toward pNPP resembling a long chain substrate was desirable. Other criteria to evaluate the effect of the mutations are the ratios of relative specific activity of pNPH and pNPO to pNPP (Table 1). Due to rather low overall activity of mutants from the third round those have not been applied in the cheese making experiments. A mutant exhibiting an appealing hydrolysis profile, was considered the most effective candidate to substitute for PGE. Mutants matching these criteria best were F91L, S163M, I245F, L302G, L305A, I529D, F91L + I245F, F91L + L305A, S163M + L302G, I245F + L302G, L302G + L305A and L302G + I529D. Therefore, one unit of each PCI_Lip mutant compared to the activity against pNPO was subjected to the making process of Feta-type brine cheese. To compare the impacts of the different mutants, samples were prepared without the addition of lipolytic enzymes, with the addition of opti-zym z10uc a commercially available PGE, and PCI_Lip WT. The results of vFFA analysis (Fig. 8) display that all applied mutants had an impact on the release of vFFA as the peak areas for C4:0 to C10:0 are higher than these in the sample without addition of a lipolytic enzyme. Even though mutants S163M and I245F exhibited significant differences in the photometrically determined hydrolysis profile, their vFFA profiles were comparable to the that of the WT. In the sensory evaluation, the addition of both mutants was perceived as more intense, sour and goat-like for both smell and taste and taste-vise the samples were also described as soapier than the one prepared with the WT. Four single and three double mutants show overall lower lipolytic activity within cheese preparation than the WT as well as the commercial PGE. The reduced activity could be seen for some of the mutants beforehand in the photometric assays, however, the overall lipolytic activity of F91L, I529D, F91L + I245F and S163M + L302G had not been seen to have strongly reduced in the prior experiments. For the double mutants L302G + L305A and L302G + I529D the overall activity in the photometric assays was lower in comparison to the WT. For the vFFA analysis the overall activity is in a similar range as the WT. The vFFA profiles of all applied mutants and the WT display the highest peak areas for hexanoic acid while the major peak area in the cheese prepared with the PGE is butyric acid. Barely any octanoic acid is released by opti-zym z10uc whereas it resembles the second highest peak area of vFFA in all cheese samples prepared with a mutant except for the one with L302G + L305A. Four single mutants seemed to be less active on butyric acid in comparison to the WT according to the peak areas. This is in contradiction to the MD simulations and is not in accordance with the photometric results. However, it could be caused by the fatty acid volatility during SPME-GC–MS detection.

**Fig. 7:**
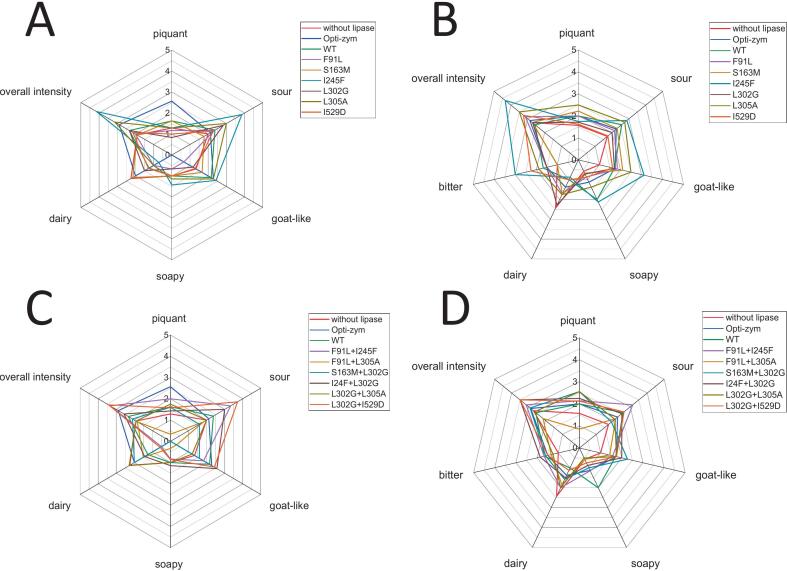
Sensory evaluation of Feta-type brine cheese produced with PCI_Lip mutants. The evaluation is based on the rating of eight panelists from 0 (no perception) to 5 (strong perception) on the given attributes, while (A) is the smell of cheese prepared with single mutants and (B) the taste of cheese prepared with single mutants, (C) is the smell of cheese prepared with double mutants and (D) the taste of cheese prepared with double mutants. For statistical evaluation see Fig. S21.

The sensory evaluations of cheeses are shown in Fig. 7. The smell of the cheese samples prepared with the addition of single mutants is still very divergent from the one described for the cheeses prepared with PGE. The samples are much lower in the piquant perception but also are described to have a soapier smell. The samples prepared with the addition of I245F and L305A are standing out in this group as their smell has been rated highest in overall intensity, sourness and most goat-like. The sample prepared with I245F had an overall more intense smell and exhibited larger peak areas in the vFFA analysis consistent with enhanced activity in photometric assays. The amount of vFFA, which majorly contribute to the piquant and goat-like flavor, for the samples prepared with F91L, L302G, L305A and I529D was much lower, which also had a milder smell than in the samples prepared with S163M and I245F. These were consistent with both the vFFA analysis and sensory evaluation, where the last two samples scored highest in all taste attributes except creaminess. The taste of the samples prepared with the other single mutants was perceived similar to the sample prepared with PGE. The soapy off-flavor of PCI_Lip due to the fortified release of long chain fatty acids could be reduced by the utilization of the engineered single mutants. All the samples except for the one prepared with I245F were rated lower in soapiness than the one with the WT. Samples prepared with four single mutants were even lower rated in soapiness than the sample prepared with PGE. Overall, the taste profile of the sample prepared with F91L was very similar to the one prepared with PGE. Here it is to point out that the addition of all single mutants except I245F as a mutant with generally increased activity led to a lower rating in soapiness than the WT. This implies that these mutants release less long chain fatty acids which was the aim of engineering PCI_Lip. This facilitates the results of MD simulations and experiments with model substrates.

**Fig. 8:**
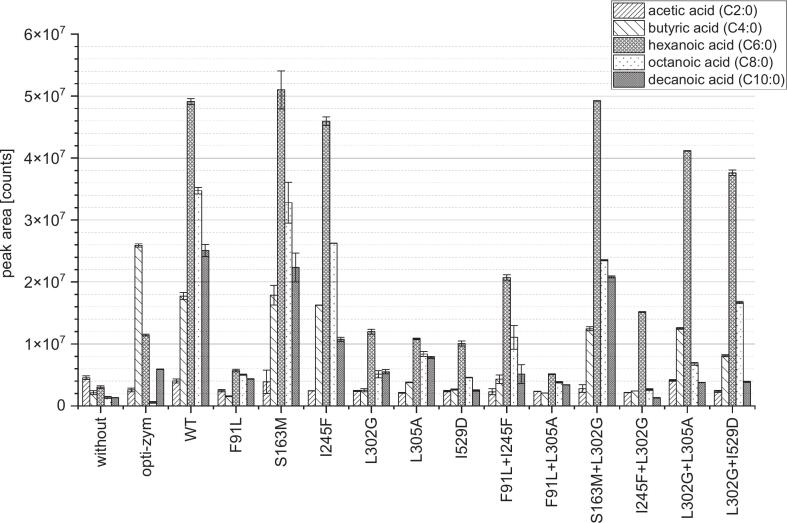
Peak areas of volatile free fatty acids measured in Feta-type brine cheese samples by SPME-GC–MS. The cheeses were produced with the addition of different lipase mutants. As a reference PGE opti-zym z10uc was used. During cheese making 1 U of lipase mutant was added to each approach, for L302G + L305A 0.5 U of lipase was added. Measurements were performed in duplicates, error bars correspond to the mean deviation. For statistical evaluation see Fig. S22.

While in the photometric experiments the overall hydrolytic activity of double mutants was lower than the one of the WT the analysis of vFFA showed that the release of vFFA of S163M + L302G, L302G + L305A and L302G + I529D in the cheese samples was in the same range as in the sample prepared with the WT. Samples prepared with double mutants are barely comparable to the smell of the sample prepared with PGE. The addition of two double mutants evoked a sourer smell than perceived in the sample prepared with PGE. These two samples were also rated highest in goat-like smell and overall intensity while simultaneously creaminess was rated lowest of all samples. In comparison with the vFFA profiles of the samples, they stand out as both exhibit a considerable release of octanoic acid. The odor perception of octanoic acid is described as rancid and goat-like (Miyazawa et al., 2009). For the sample with the addition of F91L + L305A the sensory evaluation resembles the results from the vFFA analysis. There the peak area was beyond the lowest of all samples while simultaneously the smell was described as faintly for all given attributes. Generally, the evaluation of the taste of the samples with the addition of double mutants is more similar to the sample prepared with PGE analogue to the results for the samples prepared with single mutants. Contrary to the evaluation of the smell, the taste of all samples except the one with the addition of F91L + L305A has been assessed as more piquant than the sample prepared with PGE. Compared to the sample prepared with the WT, the addition of all double mutants led to a reduction in the perception of soapiness. Also, the soapy sensation was lower than the sample prepared with PGE was rated.

The application of the second round of mutants in cheese production revealed some promising candidates to replace PGE. The addition of L302G + L305A resulted in a cheese sample with a taste which has been evaluated similar to the one prepared with PGE with even lower rating of the attributes bitter and soapy which are rather undesired in cheese. Further the double mutants S163M + L302G, I245F + L302G and L302G + I529D promote a more piquant, goat-like and intense flavor than the PGE opti-zym z10uc. As initially observed in the photometric assays, the mutation L302G seems to have a positive impact on the hydrolytic activity of PCI_Lip. This impact is transferred to the application trials as well. The combinatorial approach in mutagenesis could improve especially the taste profile of the cheese samples prepared with different double mutants, all of which carried the mutation L302G.

## 4 Conclusion

In three rounds of mutagenesis, the selectivity of the Basidiomycota derived lipase PCI_Lip was adapted to fit the requirements of animal derived PGE used in cheese production. Most mutants showed the predicted shift in the hydrolysis profiles with enhanced release of short to medium chain fatty acids and reduced hydrolysis of long chain fatty acids. Selected single and double mutants were evaluated as potential PGE substitutes in the production of Feta-type brine cheese. A vFFA analysis showed that the addition of four double mutants increased the release of short to medium chain fatty acids. Sensory evaluation confirmed their high potential as substitutes of PGE, producing a flavor comparable in piquancy to the reference cheese while markedly reducing the soapy off-flavor observed in the cheese prepared with the WT. For the production of Feta-type cheese the mutant L302G + L305A seems to be the most appropriate substitute for PGE. Cheese varieties that use PGE in the production process with a more piquant flavor such as Parmigiano Reggiano mutants S163M + L302G or L302G + I529D may be the best choice, as both provide a highly intense, piquant flavor. A dosage range of 0.5 to 1 U gave good sensory results warranting a good start for product development. Three double mutants when used in cheese with improved goat-like attributes could be identified, while the variant S163M + L302G stands out for the best overall taste, high catalytic activity toward pNPO and pNPH and improved selectivity. Kinetic characterization of these mutants underline that the substrate specificity of PCI_Lip was changed in this *in silico* guided approach for application in dairy industries. Studies on impaired mutants further validated the PCI_Lip. The importance of the lid domain for PCI_Lip-like enzymes for activity and solubility was demonstrated. These results demonstrate the potential of Basidiomycota derived lipases, especially if they are tailored. The protein engineering results combined with first application trials provide a foundation for scale up and further fine tuning of process parameters in future product development.

## CRediT authorship contribution statement

**Lea Henrich:** Writing – review & editing, Writing – original draft, Visualization, Software, Formal analysis, Data curation. **Niklas Broel:** Data curation. **Jonathan Schüler:** Data curation. **Marius Lang:** Data curation. **Binglin Li:** Writing – review & editing. **Martin Gand:** Writing – review & editing, Supervision, Conceptualization.

## Ethical statement

The authors certify that this study was conducted in strict accordance with the ethical principles outlined in the World Medical Association's Declaration of Helsinki for research involving human participants. In the context of sensory evaluation, national regulations do not require formal ethical approval, and no established ethics committee or formal documentation process is available for such studies. Despite this, the authors implemented rigorous protocols to safe­guard the rights and privacy of all participants. These protocols included ensuring voluntary participation without coercion, providing comprehensive information about the study's aims, procedures, and potential risks, obtaining verbally informed consent from each participant, ensuring that no participant data was disclosed without prior consent, and allowing participants the freedom to withdraw from the study at any point without prejudice.

## Funding

National Natural Science Foundation of China for Young Scholars (grant numbers 22108227), Shaanxi Fundamental Science Research Project for Chemistry & Biology (Grant No. 22JHQ085). Lea Henrich and Martin Gand were funded by the 10.13039/501100001659Deutsche Forschungsgemeinschaft (DFG, German Research Foundation) – Project number 514527182.

## Declaration of competing interest

The authors declare that they have no known competing financial interests or personal relationships that could have appeared to influence the work reported in this paper.

## Data Availability

No data was used for the research described in the article.

## Appendix A Supplementary data

Supplementary material: The supplementary material consists of 22 figures, three tables, and a detailed description of specific methods. Image 1
