# Supplementary material for: Computational and experimental engineering of a Pleurotus citrinopileatus lipases: Structural insights and functional optimization to adapt the hydrolytic profile for cheese applications
Source: Food Chem X. 2026 Feb 6;34:103639. doi: 10.1016/j.fochx.2026.103639 (PMC12907230; doi:10.1016/j.fochx.2026.103639)
Supplement: Supplementary file 1 — Supplementary material: The supplementary material consists of 22 figures, three tables, and a detailed description of specific methods. [file mmc1.docx]

Computational and Experimental Engineering of a *Pleurotus citrinopileatus* Lipases: Structural Insights and Functional Optimization to Adapt the Hydrolytic Profile for Cheese Applications

Lea Henrich^a^, Niklas Broel^a^, Jonathan Schüler^a^, Marius Lang^a^, Binglin Li^a,b,c,d,*^, Martin Gand^a,*^

^a^ Institute of Food Chemistry and Food Biotechnology, Justus Liebig University Giessen, Germany

^b^ Institute of Food Science and Technology, Chinese Academy of Agricultural Sciences, Beijing, China

^c^ College of Food Science and Engineering, Northwest University, Shaanxi, China

^d^ Cangzhou Academy of Agriculture and Forestry Sciences, Cangzhou, China

* Corresponding authors


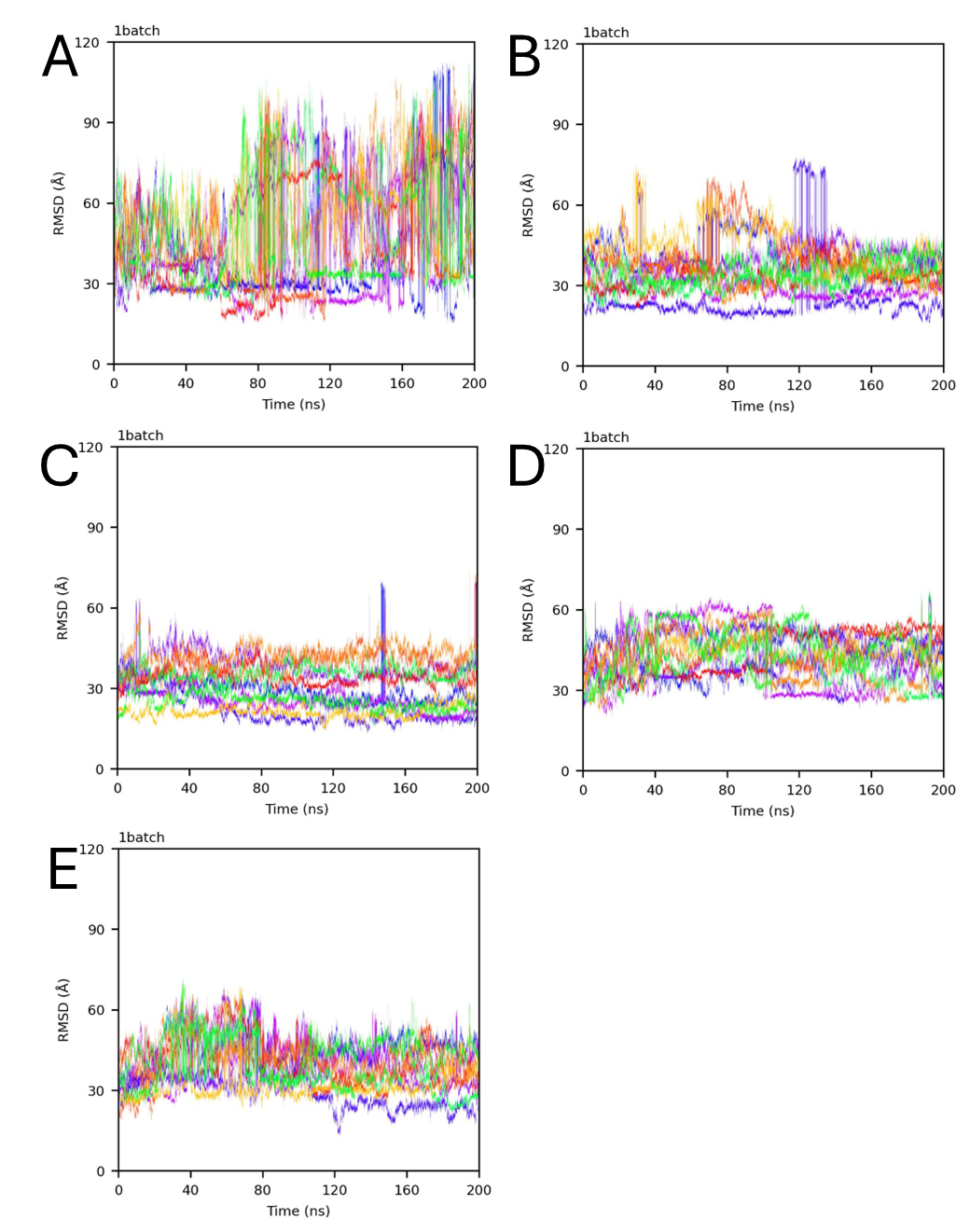


**Fig S1.** Time evolution of RMSD for the distance between triglycerides and the active center (S213). (A) C4:0; (B) C10:0; (C) C16:0; (D) C18:0; (E) C18:1. MD simulations were repeated three times for each case. Only the original data from the first batch is presented to reduce the number of figures used.


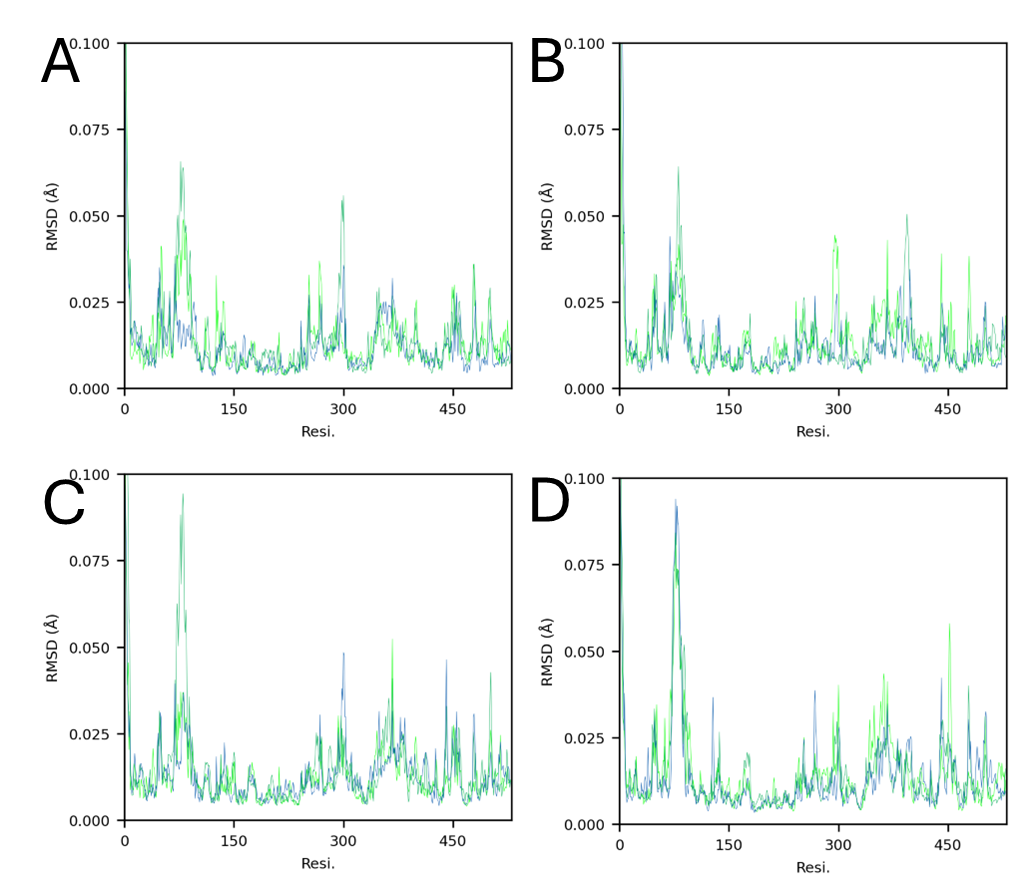


**Fig. S2.** RMSD of each residue when (A) C4:0; (B) C10:0; (C) C18:0; and (D) C18:1 were used in the MD simulations, respectively.


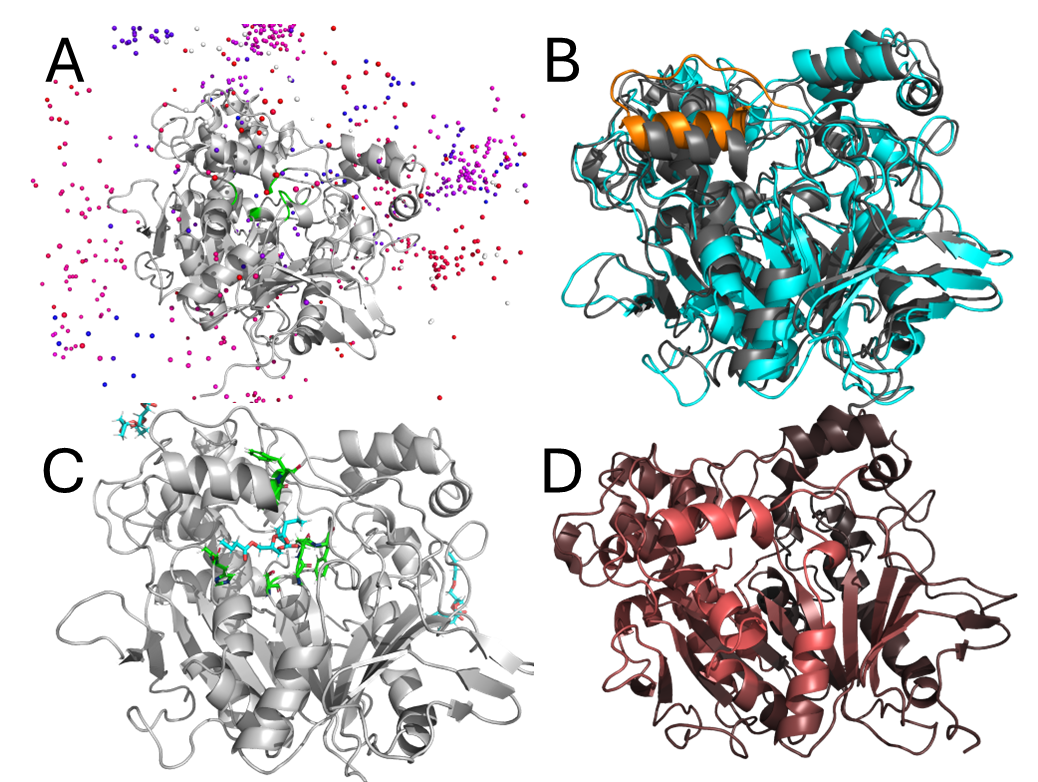


**Fig. S3.** Molecular dynamics simulation analysis of PCI_Lip (C4:0). (A) Trajectories of the glycerol center of triglycerides are displayed. Only trajectories of the substrates, which could enter the active pocket were shown. Other molecules were hidden for easier visualization. The catalytic pocket was colored as green. (B) Alignment analysis of the protein structure change. The typical structure was shown by cyan and confirmed when the minimal distance was obtained between any carbonyl carbon of triglycerides and the hydroxyl group of S213 of PCI_Lip, the lid domain was colored in orange. The initial structure was colored gray. (C) The snapshot of the typical structure, including protein (gray) and triglycerides (cyan). The catalytic pocket was colored in green. (D) Statistics of average interaction frequencies of each residue with triglycerides. The values of the residues with the strongest affinity were set to 100% and 0%, with the corresponding colors assigned as red and black, respectively.


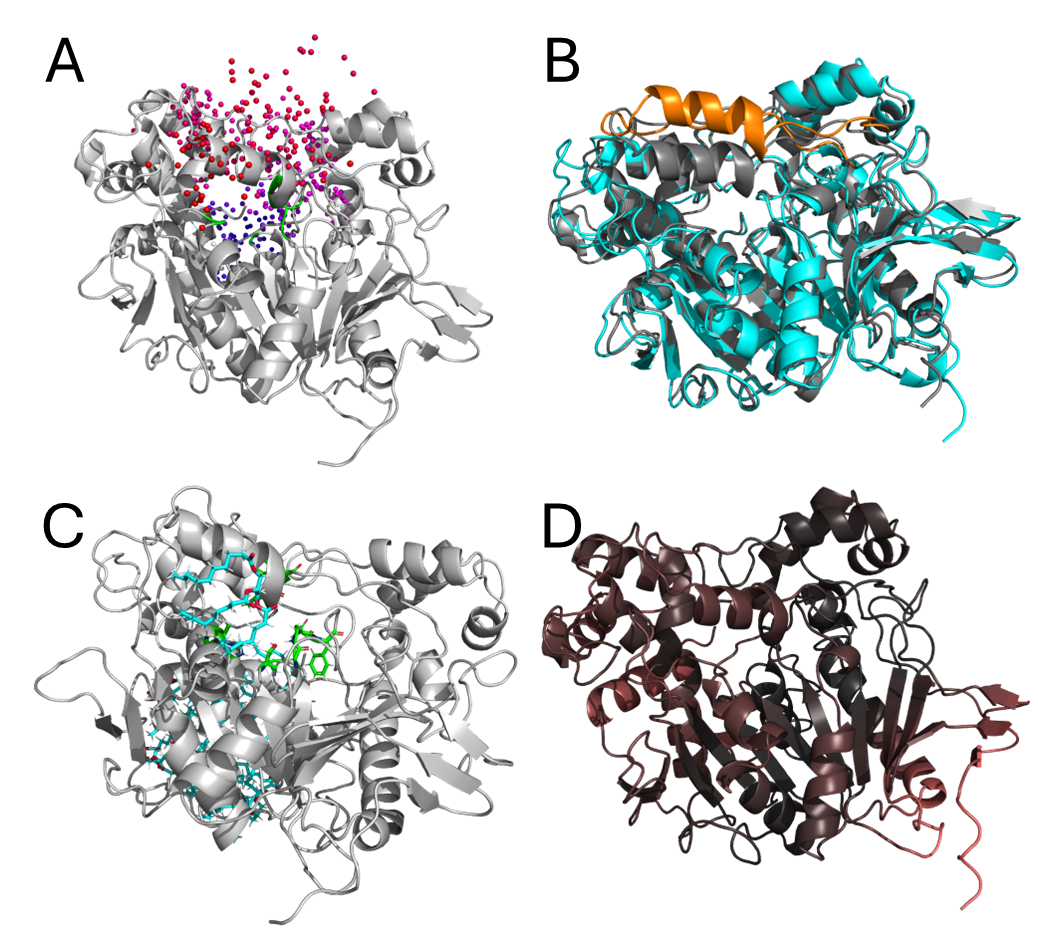
 **Fig. S4.** Molecular dynamics simulation analysis of PCI_Lip (C10:0). (A) Trajectories of the glycerol center of triglycerides are displayed. Only trajectories of the substrates, which could enter the active pocket were shown. Other molecules were hidden for easier visualization. The catalytic pocket was represented as green. (B) Alignment analysis of the protein structure change. The typical structure was shown by cyan and confirmed when the minimal distance was obtained between any carbonyl carbon of triglycerides and the hydroxyl group of S213 of PCI_Lip, the lid domain was colored in orange. The initial structure was colored gray. (C) The snapshot of the typical structure, including protein (gray) and triglycerides (cyan). The catalytic pocket was colored in green. (D) Statistics of average interaction frequencies of each residue with triglycerides. The values of the residues with the strongest affinity were set to 100% and 0%, with the corresponding colors assigned as red and black, respectively.


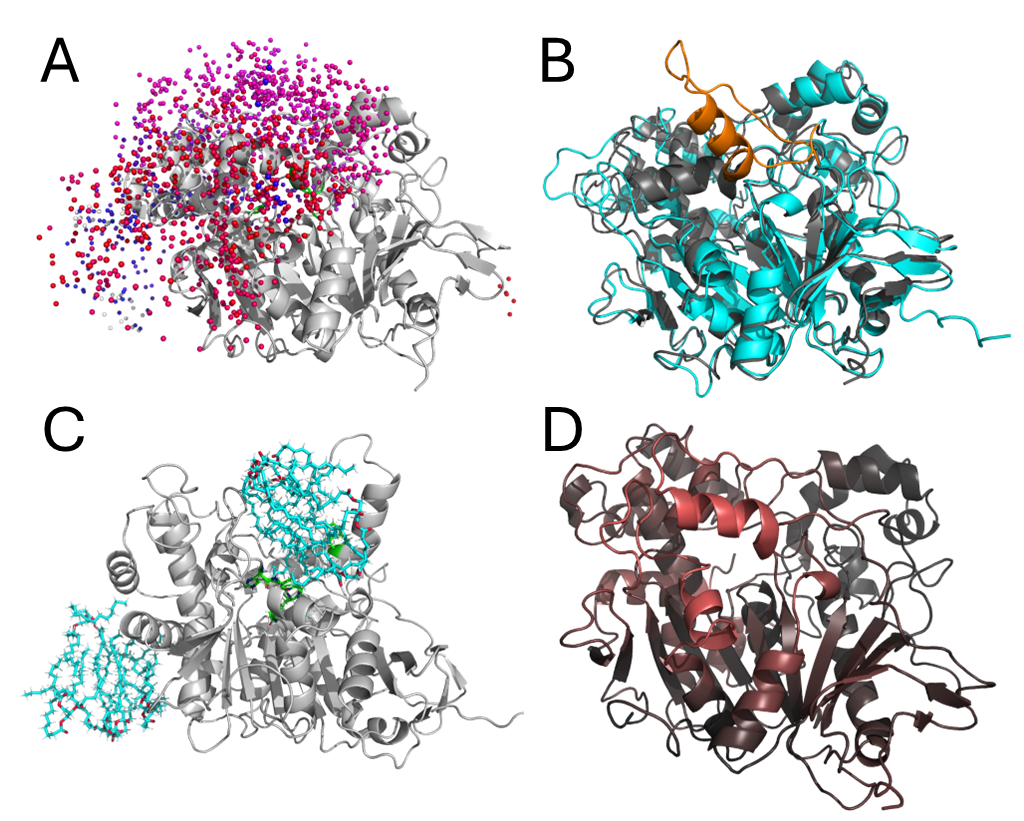
 **Fig. S5.** Molecular dynamics simulation analysis of PCI_Lip (C16:0). (A) Trajectories of the glycerol center of triglycerides are displayed. Only trajectories of the substrates, which could enter the active pocket were shown. Other molecules were hidden for easier visualization. The catalytic pocket was represented as green. (B) Alignment analysis of the protein structure change. The typical structure was shown by cyan and confirmed when the minimal distance was obtained between any carbonyl carbon of triglycerides and the hydroxyl group of S213 of PCI_Lip, the lid domain was colored in orange. The initial structure was colored gray. (C) The snapshot of the typical structure, including protein (gray) and triglycerides (cyan). The catalytic pocket was colored in green. (D) Statistics of average interaction frequencies of each residue with triglycerides. The values of the residues with the strongest affinity were set to 100% and 0%, with the corresponding colors assigned as red and black, respectively.


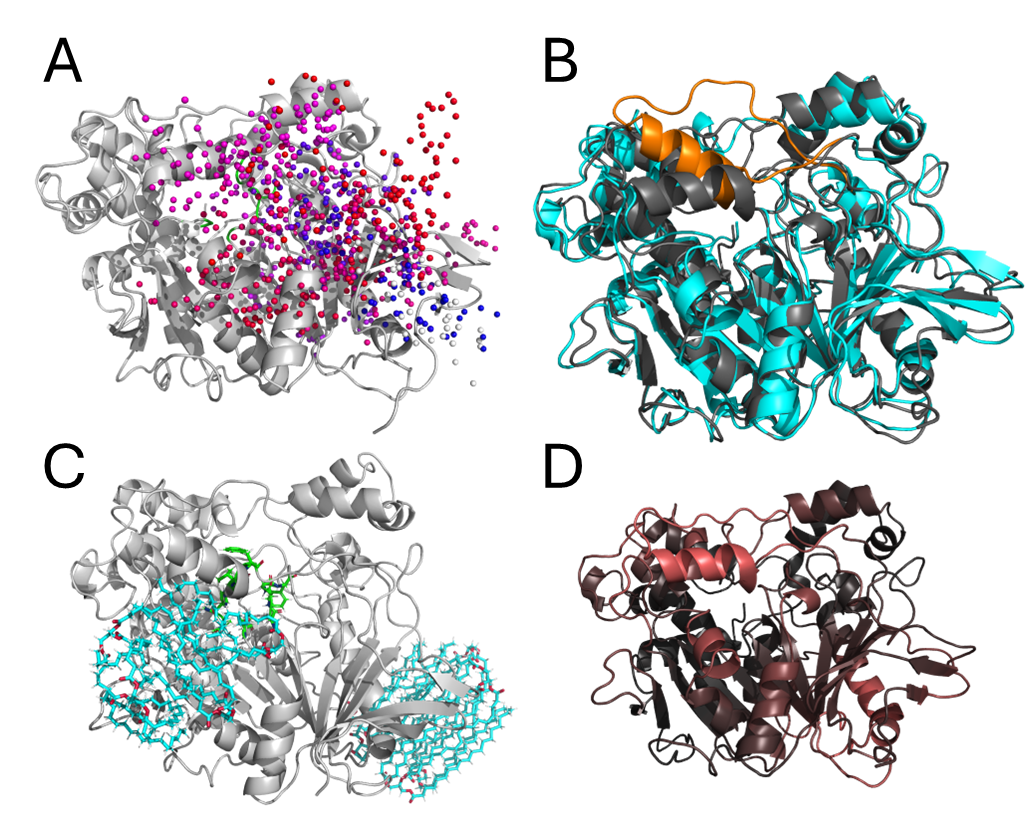


**Fig. S6.** Molecular dynamics simulation analysis of PCI_Lip (C18:0). (A) Trajectories of the glycerol center of triglycerides are displayed. Only trajectories of the substrates, which could enter the active pocket were shown. Other molecules were hidden for easier visualization. The catalytic pocket was represented as green. (B) Alignment analysis of the protein structure change. The typical structure was shown by cyan and confirmed when the minimal distance was obtained between any carbonyl carbon of triglycerides and the hydroxyl group of S213 of PCI_Lip, the lid domain was colored in orange). The initial structure was colored gray. (C) The snapshot of the typical structure, including protein (gray) and triglycerides (cyan). The catalytic pocket was colored in green. (D) Statistics of average interaction frequencies of each residue with triglycerides. The values of the residues with the strongest affinity were set to 100% and 0%, with the corresponding colors assigned as red and black, respectively.


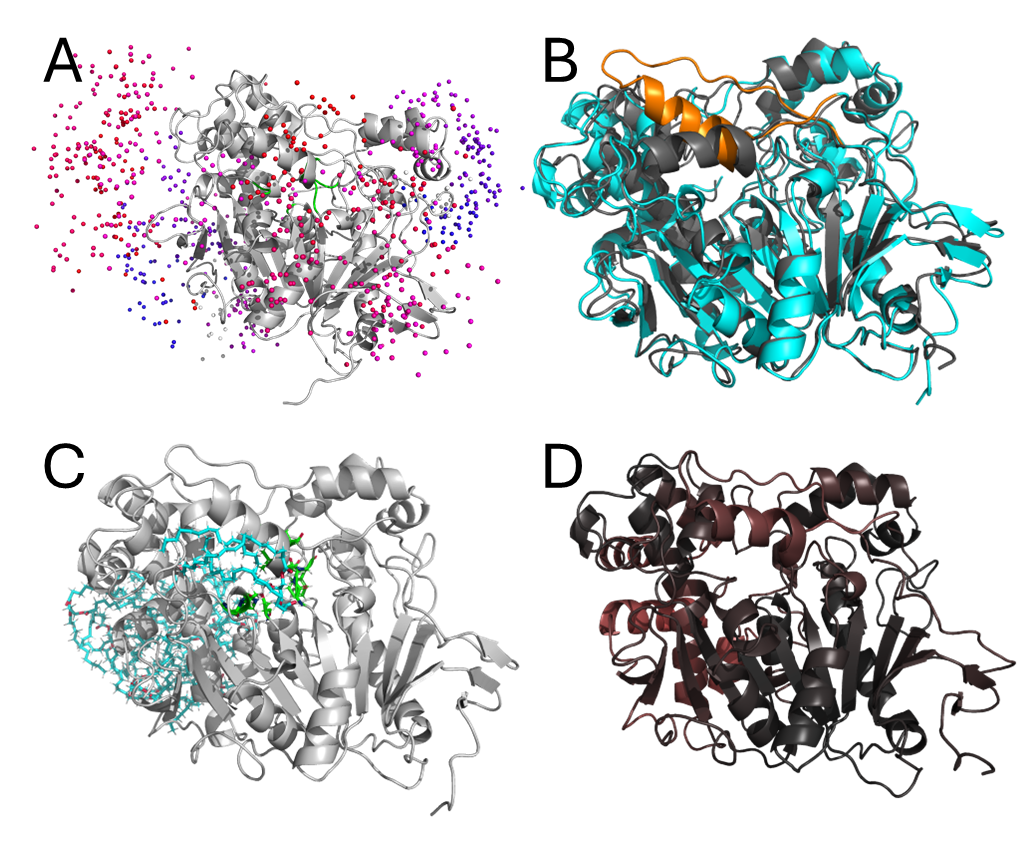
 **Fig. S7.** Molecular dynamics simulation analysis of PCI_Lip (C18:1). (A) Trajectories of the glycerol center of triglycerides are displayed. Only trajectories of the substrates, which could enter the active pocket were shown. Other molecules were hidden for easier visualization. The catalytic was represented as green. (B) Alignment analysis of the protein structure change. The typical structure was shown by cyan and confirmed when the minimal distance was obtained between any carbonyl carbon of triglycerides and the hydroxyl group of S213 of PCI_Lip, the lid domain was colored in orange. The initial structure was colored gray. (C) The snapshot of the typical structure, including protein (gray) and triglycerides (cyan). The catalytic pocket was colored in green. (D) Statistics of average interaction frequencies of each residue with triglycerides. The values of the residues with the strongest affinity were set to 100% and 0%, with the corresponding colors assigned as red and black, respectively.


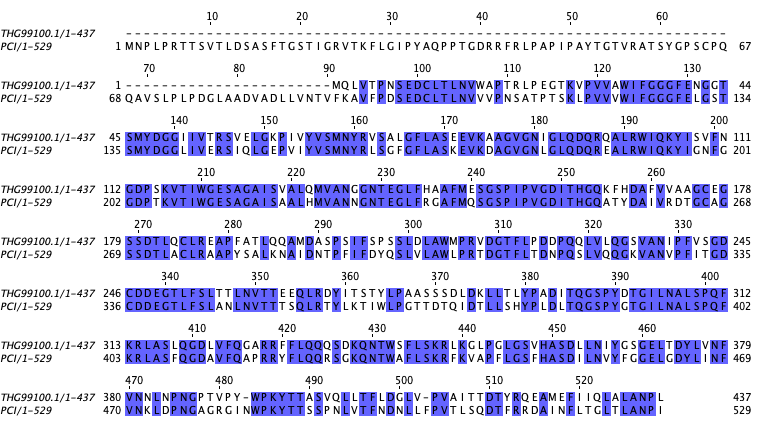


**Fig. S8.** Protein sequence alignment of PCE_Lip and PCI_Lip.


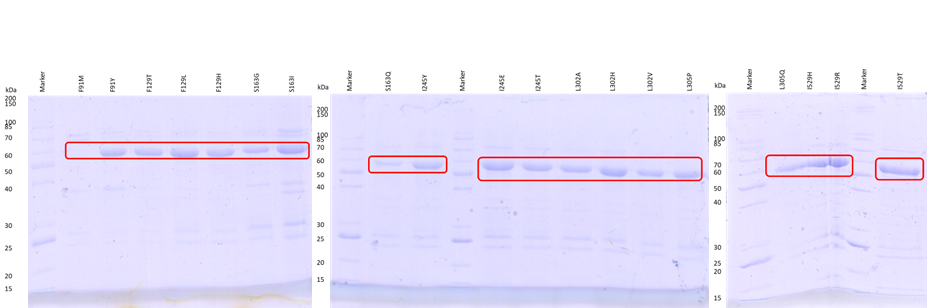


**Fig. S9.** SDS-PAGE gels of PCI_Lip single mutants created during this study with (framed in red) after expression and purification. M refers to the marker Unstained Protein Marker Broad Range (NEB, Germany). The molecular mass of PCI_Lip with His_6_-tag is approximately 62 kDa.

(1)
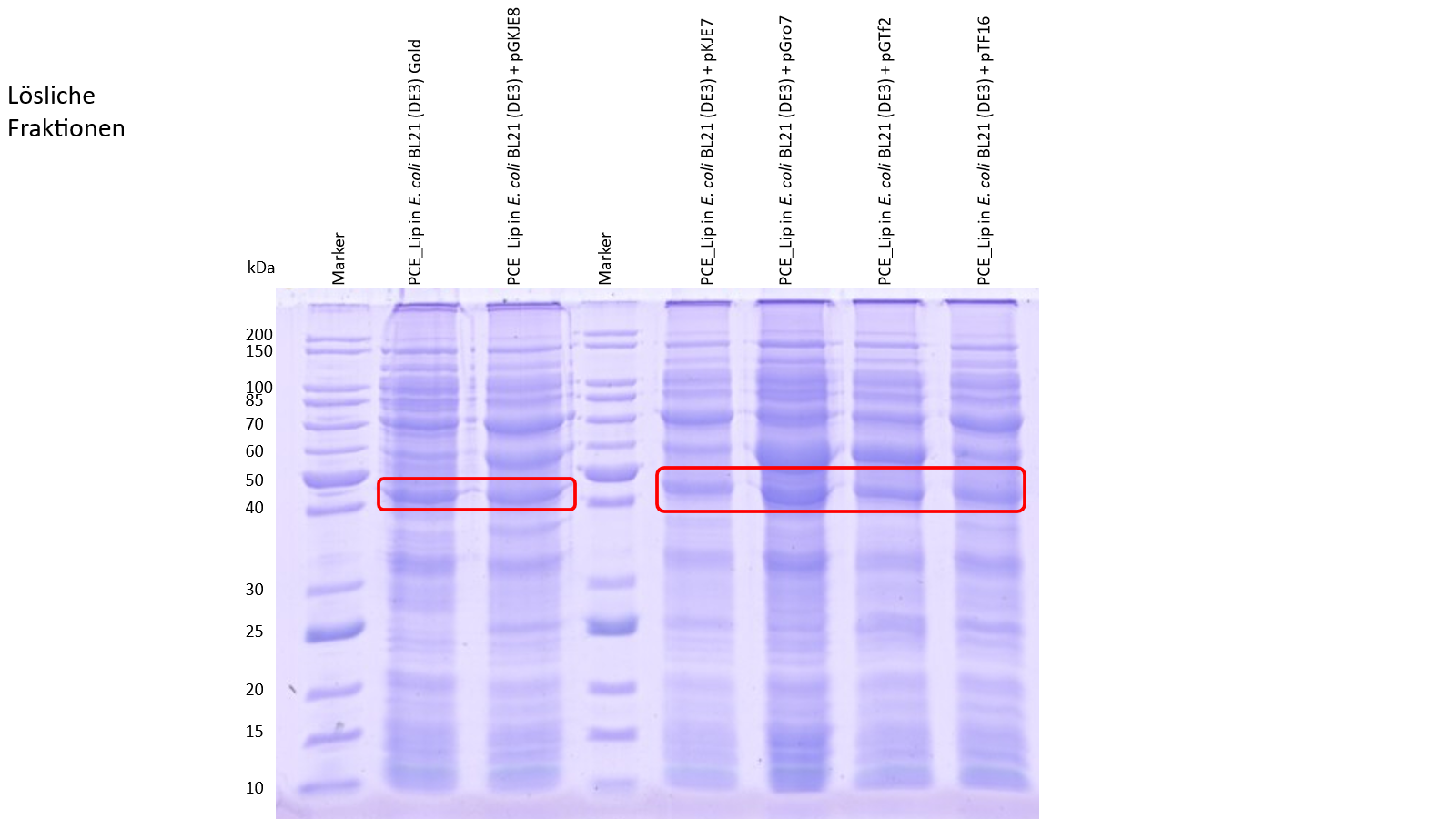
(2)
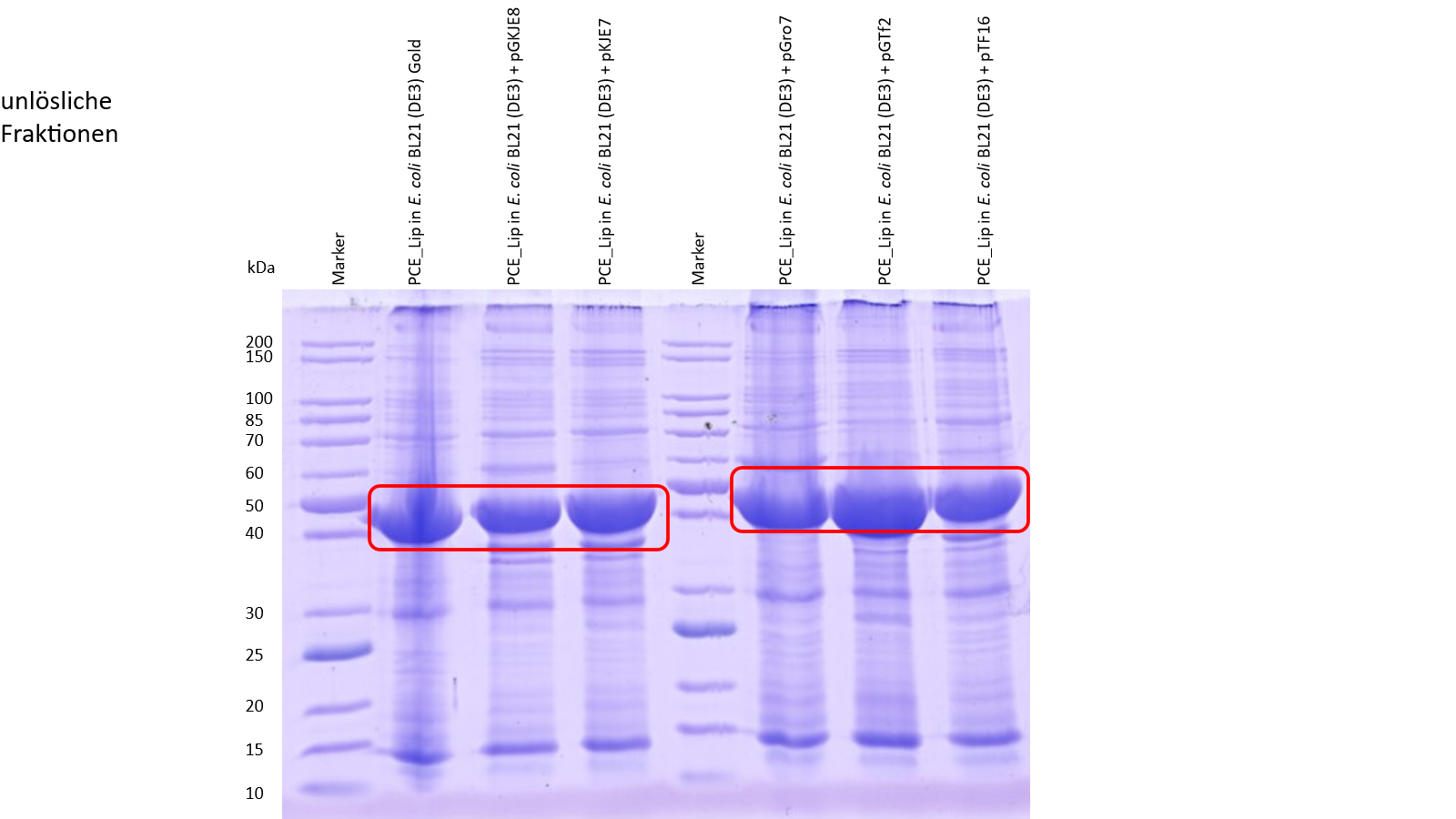


**Fig. S10.** SDS-PAGE gels of PCE_Lip expression using different expression systems and chaperones. As a marker the Unstained Protein Marker Broad Range (NEB, Germany) is used. The molecular mass of PCE_Lip with His_6_-tag is approximately 48 kDa. (1) Soluble protein fraction after cultivation, no overexpression of PCE_Lip is observed. (2) Insoluble protein fraction after cultivation with over expression bands of PCE_Lip (framed in red).


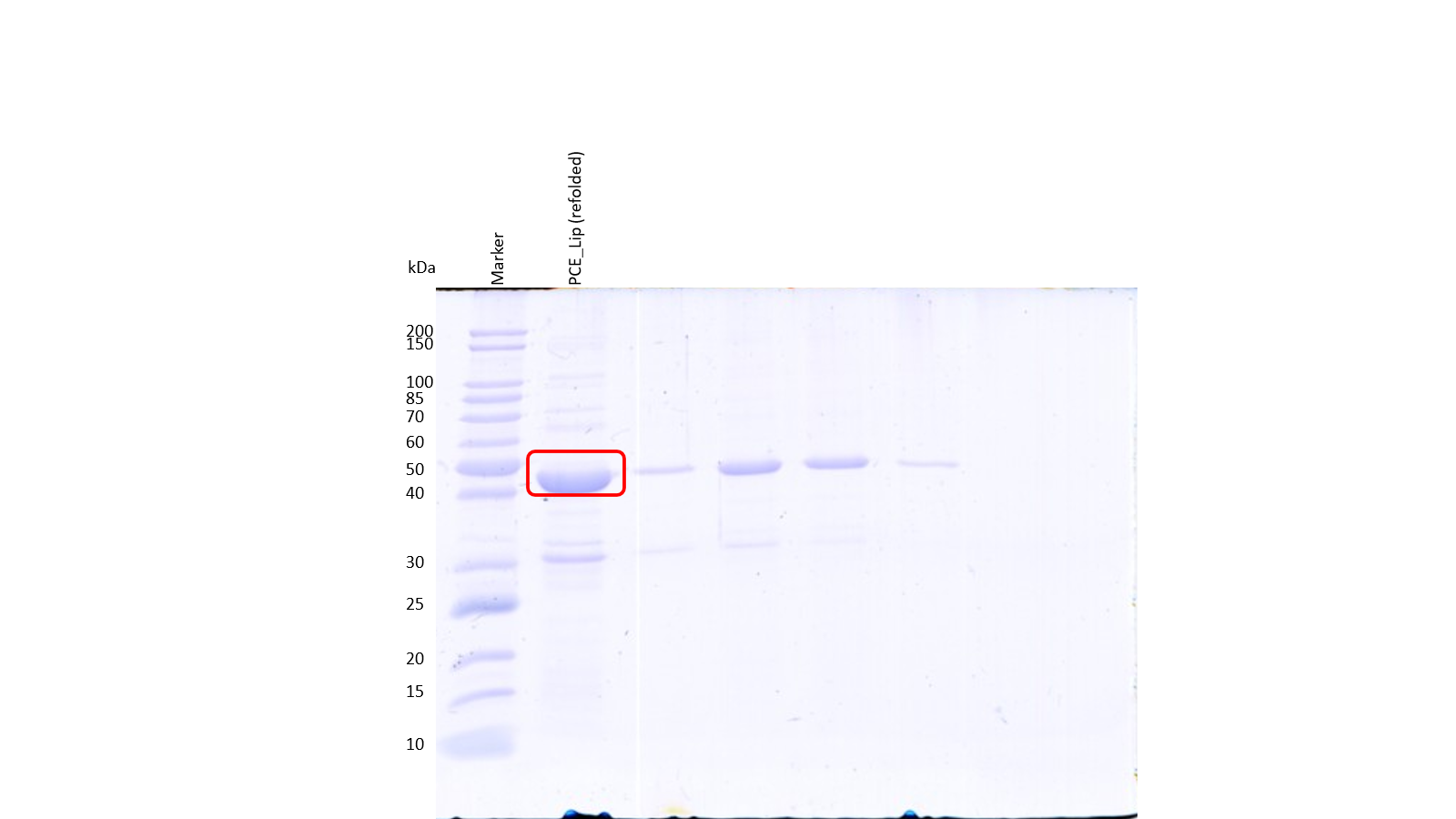

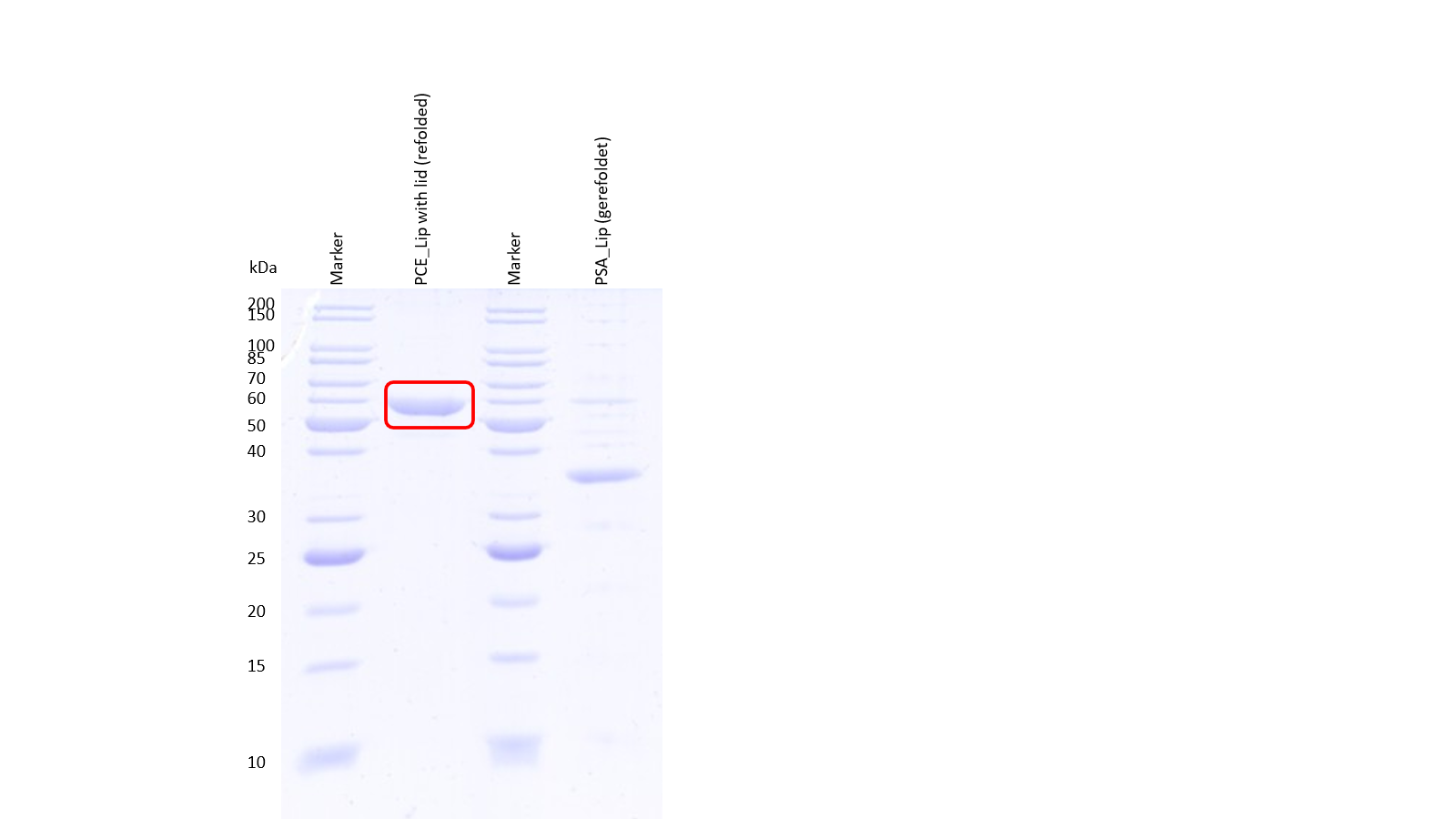


**Fig. S11.** SDS-PAGE gels of PCE_Lip and PCE_Lip with lid (framed in red) after expression and refolding. Marker refers to the marker Unstained Protein Marker Broad Rang (NEB, Germany). The molecular mass of PCE_Lip with His_6_-tag is approximately 48 kDa and of PCE_Lip with lid and His_6_-tag is approximately 56 kDa.


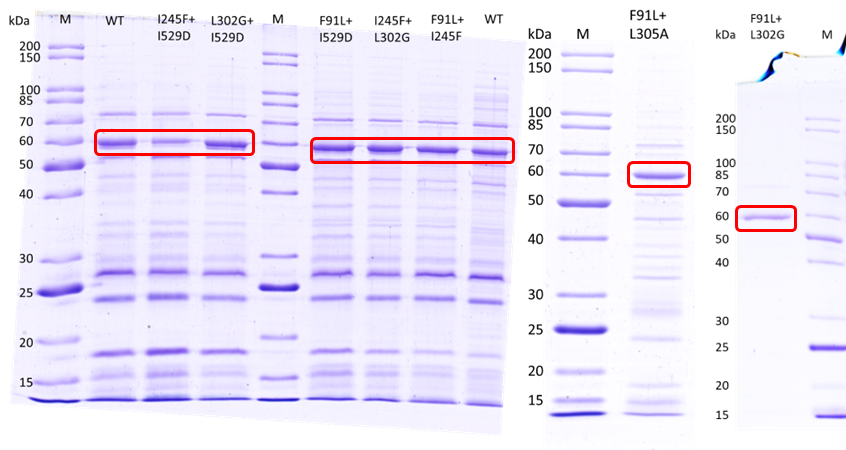


**Fig. S12.** SDS-PAGE gels of PCI_Lip double mutants (framed in red) after expression and purification. M refers to the marker Unstained Protein Marker Broad Range (NEB, Germany). As a reference, the WT has been run beside the mutants. The molecular mass of PCI_Lip with His_6_-tag is approximately 62 kDa.


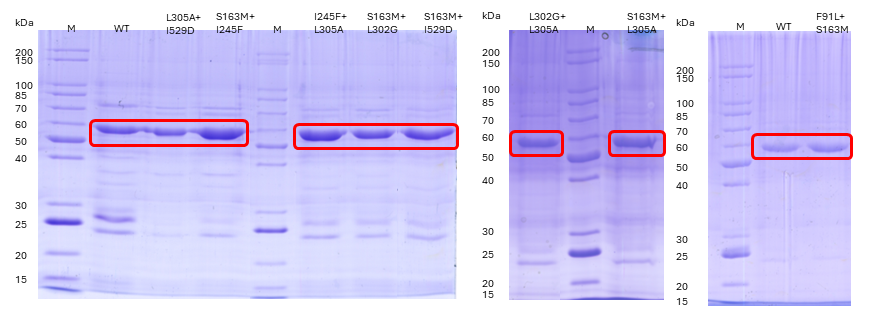


**Fig. S13.** SDS-PAGE gels of PCI_Lip double mutants (framed in red) after expression and purification. M refers to the marker Unstained Protein Marker Broad Range (NEB, Germany). As a reference, the WT has been run beside the mutants. The molecular mass of PCI_Lip with His_6_-tag is approximately 62 kDa.


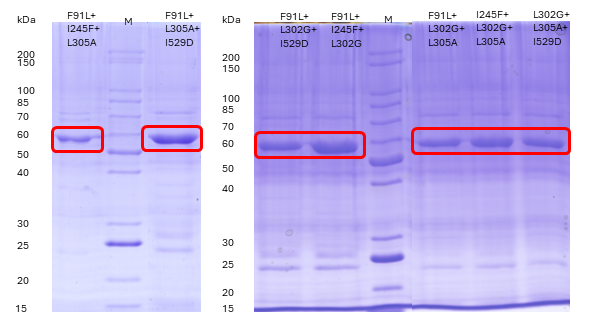


**Fig. S14.** SDS-PAGE gels of PCI_Lip triple mutants (framed in red) after expression and purification. M refers to the marker Unstained Protein Marker Broad Range (NEB, Germany). The molecular mass of PCI_Lip with His_6_-tag is approximately 62 kDa.

**Table S1.** Primers for the generation of mutants used in this study with respective melting temperatures (*T*_M_). Mutants marked with * have been characterized by Broel et al. Fw = forward, rv = reverse

| Mutation site | Orientation | Sequence (5' -> 3') |  | *T*_M_ [°C] | |
| --- | --- | --- | --- | --- | --- |
| F91M | fw | GAATACCGTTATGAAAGCCGTGTTTCCGG | | 71 |  |
|  | rv | CCGGAAACACGGCTTTCATAACGGTATTC | | 71 |  |
| F91Y | fw | GAATACCGTTTATAAAGCCGTGTTTCCGG | | 69 |  |
|  | rv | CCGGAAACACGGCTTTATAAACGGTATTC | | 69 |  |
| F91G | fw | GAATACCGTTGGCAAAGCCGTGTTTCCGG | | 76 |  |
|  | rv | CACGGCTTTGCCAACGGTATTCACCAGCAG | | 77 |  |
| F91H* | fw | GAATACCGTTCATAAAGCCGTGTTTCCGG | | 71 |  |
|  | rv | GAATACCGTTCATAAAGCCGTGTTTCCGG | | 71 |  |
| F91L* | fw | GAATACCGTTCTGAAAGCCGTGTTTCCGG | | 73 |  |
|  | rv | CACGGCTTTCAGAACGGTATTCACCAGCAG | | 75 |  |
| F91N* | fw | GAATACCGTTAACAAAGCCGTGTTTCCGG | | 72 |  |
|  | rv | CACGGCTTTGTTAACGGTATTCACCAGCA | | 73 |  |
| F91T* | fw | GAATACCGTTACCAAAGCCGTGTTTCCGG | | 73 |  |
|  | rv | CACGGCTTTGGTAACGGTATTCACCAGCAG | | 75 |  |
| F129T | fw | GGCGGCACGGAACTGGGTAGTACCAGCATG | | 80 |  |
|  | rv | CATGCTGGTACTACCCAGTTCCGTGCCGCC | | 80 |  |
| F129L | fw | GGCGGCCTGGAACTGGGTAGTACCAGCATG | | 80 |  |
|  | rv | CATGCTGGTACTACCCAGTTCCAGGCCGCC | | 80 |  |
| F129H | fw | GGCGGCCATGAACTGGGTAGTACCAGCATG | | 78 |  |
|  | rv | CATGCTGGTACTACCCAGTTCATGGCCGCC | | 78 |  |
| F129A* | fw | GGCGGCGCGGAACTGGGTAGTACCAGCATG | | 82 |  |
|  | rv | CCAGTTCCGCGCCGCCGCCAAAAATCCACACC | | 84 |  |
| F129C* | fw | GGCGGCTGCGAACTGGGTAGTACCAGCATG | | 80 |  |
|  | rv | CCAGTTCGCAGCCGCCGCCAAAAATCCACACC | | 82 |  |
| F129Q* | fw | GGCGGCCAGGAACTGGGTAGTACCAGCATG | | 80 |  |
|  | rv | CCAGTTCCTGGCCGCCGCCAAAAATCCACACC | | 82 |  |
| F129R* | fw | GGCGGCCGCGAACTGGGTAGTACCAGCATG | | 82 |  |
|  | rv | CCAGTTCGCGGCCGCCGCCAAAAATCCACACC | | 84 |  |
| S163H | fw | GAATTATCGTCTGCATGGTTTTGGCTTTCTGG C | | 73 |  |
|  | rv | GCCAAAACCATGCAGACGATAATTCATGCTAA C | | 71 |  |
| S163M | fw | GAATTATCGTCTGATGGGTTTTGGCTTTCTGG C | | 72 |  |
|  | rv | GCCAAAACCCATCAGACGATAATTCATGCTAA C | | 71 |  |
| S163V | fw | GAATTATCGTCTGGTGGGTTTTGGCTTTCTGG C | | 74 |  |
|  | rv | GCCAAAACCCACCAGACGATAATTCATGCTAA C | | 74 |  |
| S163G | fw | GAATTATCGTCTGGGCGGTTTTGGCTTTCTGGC | | 76 |  |
|  | rv | GCCAGAAAGCCAAAACCGCCCAGACGATAATTC | | 76 |  |
| S163I | fw | GAATTATCGTCTGATTGGTTTTGGCTTTCTGGC | | 71 |  |
|  | rv | GCCAGAAAGCCAAAACCAATCAGACGATAATTC | | 71 |  |
| S163Q | fw | GAATTATCGTCTGCAGGGTTTTGGCTTTCTGGC | | 74 |  |
|  | rv | GCCAGAAAGCCAAAACCCTGCAGACGATAATTC | | 74 |  |
| I245Y | fw | CTTTATGCAGAGTGGTAGCCCGTATCCGGTG | | 75 |  |
|  | rv | CACCGGATACGGGCTACCACTCTGCATAAAG | | 75 |  |
| I245E | fw | CTTTATGCAGAGTGGTAGCCCGGAACCGGTG | | 77 |  |
|  | rv | CACCGGTTCCGGGCTACCACTCTGCATAAAG | | 77 |  |
| I245T | fw | CTTTATGCAGAGTGGTAGCCCGACCCCGGTG | | 79 |  |
|  | rv | CACCGGGGTCGGGCTACCACTCTGCATAAAG | | 77 |  |
| I245W | fw | CTTTATGCAGAGTGGTAGCCCGATTCCGGTG | | 76 |  |
|  | rv | CATGGGTAATATCGCCCACCGGAATCGGGC | | 78 |  |
| I245F* | fw | GCAGAGTGGTAGCCCGTTTCCGGTGGGCG | | 82 |  |
|  | rv | GTAATATCGCCCACCGGAAACGGGCTACCAC | | 87 |  |
| L300R* | fw | CCGTTTATTTTTGATTACCAGAGCCGTGTTCTGG | | 72 |  |
|  | rv | CGGCAGCCATGCCAGAACACGGCTCTGG | | 82 |  |
| L300P* | fw | CCGTTTATTTTTGATTACCAGAGCCCGGTTCTGG | | 74 |  |
|  | rv | CGGCAGCCATGCCAGAACCGGGCTCTGG | | 84 |  |
| L300I* | fw | CCGTTTATTTTTGATTACCAGAGCATTGTTCTGG | | 69 |  |
|  | rv | CGGCAGCCATGCCAGAACAATGCTCTGG | | 78 |  |
| L302A | fw | GAGCCTGGTTGCGGCATGGCTGCCGCGTAC | | 84 |  |
|  | rv | GTACGCGGCAGCCATGCCGCAACCAGGCTC | | 84 |  |
| L302H | fw | GAGCCTGGTTCATGCATGGCTGCCGCGTAC | | 81 |  |
|  | rv | GTACGCGGCAGCCATGCATGAACCAGGCTC | | 81 |  |
| L302V | fw | GAGCCTGGTTGTGGCATGGCTGCCGCGTAC | | 83 |  |
|  | rv | GTACGCGGCAGCCATGCCACAACCAGGCTC | | 83 |  |
| L302G* | fw | GAGCCTGGTTGGCGCATGGCTGCCGCGTAC | | 84 |  |
|  | rv | GCAGCCATGCGCCAACCAGGCTCTGGTAATC | | 80 |  |
| L302P* | fw | GAGCCTGGTTCCGGCATGGCTGCCGCGTAC | | 84 |  |
|  | rv | GCAGCCATGCCGGAACCAGGCTCTGGTAATC | | 80 |  |
| L305P | fw | CTGGTTCTGGCATGGCCGCCGCGTAC | | 81 |  |
|  | rv | GTACGCGGCGGCCATGCCAGAACCAG | | 81 |  |
| L305Q | fw | CTGGTTCTGGCATGGCAGCCGCGTAC | | 79 |  |
|  | rv | GTACGCGGCTGCCATGCCAGAACCAG | | 79 |  |
| L305A* | fw | CTGGTTCTGGCATGGCTGCCGCGTAC | | 79 |  |
|  | rv | GAAAGGTACCATCTGTACGCGGCGCCCATG | | 78 |  |
| L305M* | fw | CTGGTTCTGGCATGGATGCCGCGTACAG | | 77 |  |
|  | rv | GAAAGGTACCATCTGTACGCGGCATCCATGCC | | 77 |  |
| L305N* | fw | CTGGTTCTGGCATGGAACCCGCGTACAG | | 77 |  |
|  | rv | GAAAGGTACCATCTGTACGCGGGTTCCATGCC | | 77 |  |
| L305R* | fw | CTGGTTCTGGCATGGCGTCCGCGTACAG | | 79 |  |
|  | rv | GAAAGGTACCATCTGTACGCGGACGCCATGCC | | 79 |  |
| L305H* | fw | CTGGTTCTGGCATGGCATCCGCGTACAG | | 77 |  |
|  | rv | GAAAGGTACCATCTGTACGCGGATGCCATGCC | | 77 |  |
| L305Y* | fw | CTGGTTCTGGCATGGTATCCGCGTACAG | | 75 |  |
|  | rv | GAAAGGTACCATCTGTACGCGGATACCATGCC | | 75 |  |
| I529H | fw | CAAATCCGCATTAAGCGGCCGCACTCGAGCA | | 80 |  |
|  | rv | GTGCTCGAGTGCGGCCGCTTAATGCGGATTTG | | 80 |  |
| I529R | fw | CAAATCCGCGTTAAGCGGCCGCACTCGAGCAC | | 81 |  |
|  | rv | GTGCTCGAGTGCGGCCGCTTAACGCGGATTTG | | 81 |  |
| I529T | fw | CAAATCCGACCTAAGCGGCCGCACTCGAGCAC | | 81 |  |
|  | rv | CAAATCCGACCTAAGCGGCCGCACTCGAGCAC | | 81 |  |
| I529E | fw | CAAATCCGGAATAAGCGGCCGCACTCGAGCA C | | 80 |  |
|  | rv | CCGCTTATTCCGGATTTGCCAGGGTCAGGCC | | 80 |  |
| I529A* | fw | CAAATCCGGCGTAAGCGGCCGCACTCGAGCAC | | 83 |  |
|  | rv | CCGCTTACGCCGGATTTGCCAGGGTCAGGCC | | 83 |  |
| I529D* | fw | CAAATCCGGATTAAGCGGCCGCACTCGAGCA C | | 79 |  |
|  | rv | CCGCTTAATCCGGATTTGCCAGGGTCAGGCC | | 80 |  |
| I529G* | fw | CAAATCCGGGCTAAGCGGCCGCACTCGAGCAC | | 83 |  |
|  | rv | CCGCTTAGCCCGGATTTGCCAGGGTCAGGCC | | 83 |  |
| I529W* | fw | GACCCTGGCAAATCCGTGGTAAGCGGCCGC | | 82 |  |
|  | rv | CTCGAGTGCGGCCGCTTACCACGGATTTGC | | 80 |  |
| L302G+L305A | fw | GAGCCTGGTTGGCGCATGGGCGCCGCGT AC | | 86 |  |
|  | rv | GTACGCGGTGCCCATGCACCAACCAGG | | 80 |  |


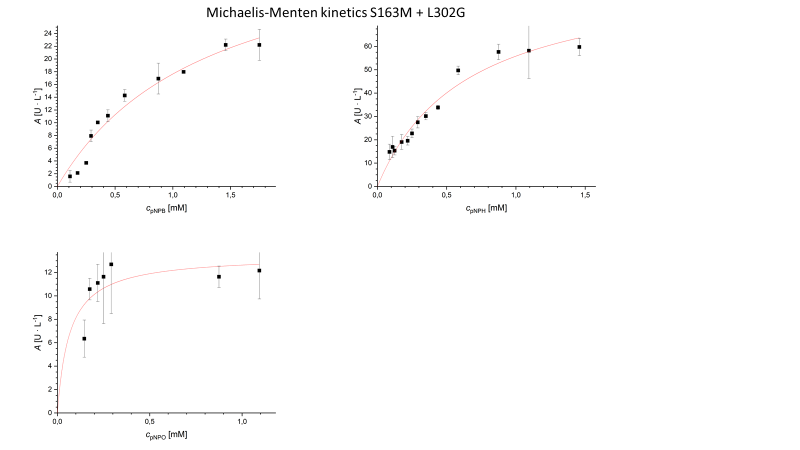


**Fig. S15.** Saturation curves of double mutant S163M+L302G against pNPB, pNPH and pNPO. Obtained data for the activity were fitted in OriginPro 2023 based on the Michaelis-Menten equation. Activity at all concentrations was measured in triplicates. Error bars represent the standard deviation.

.
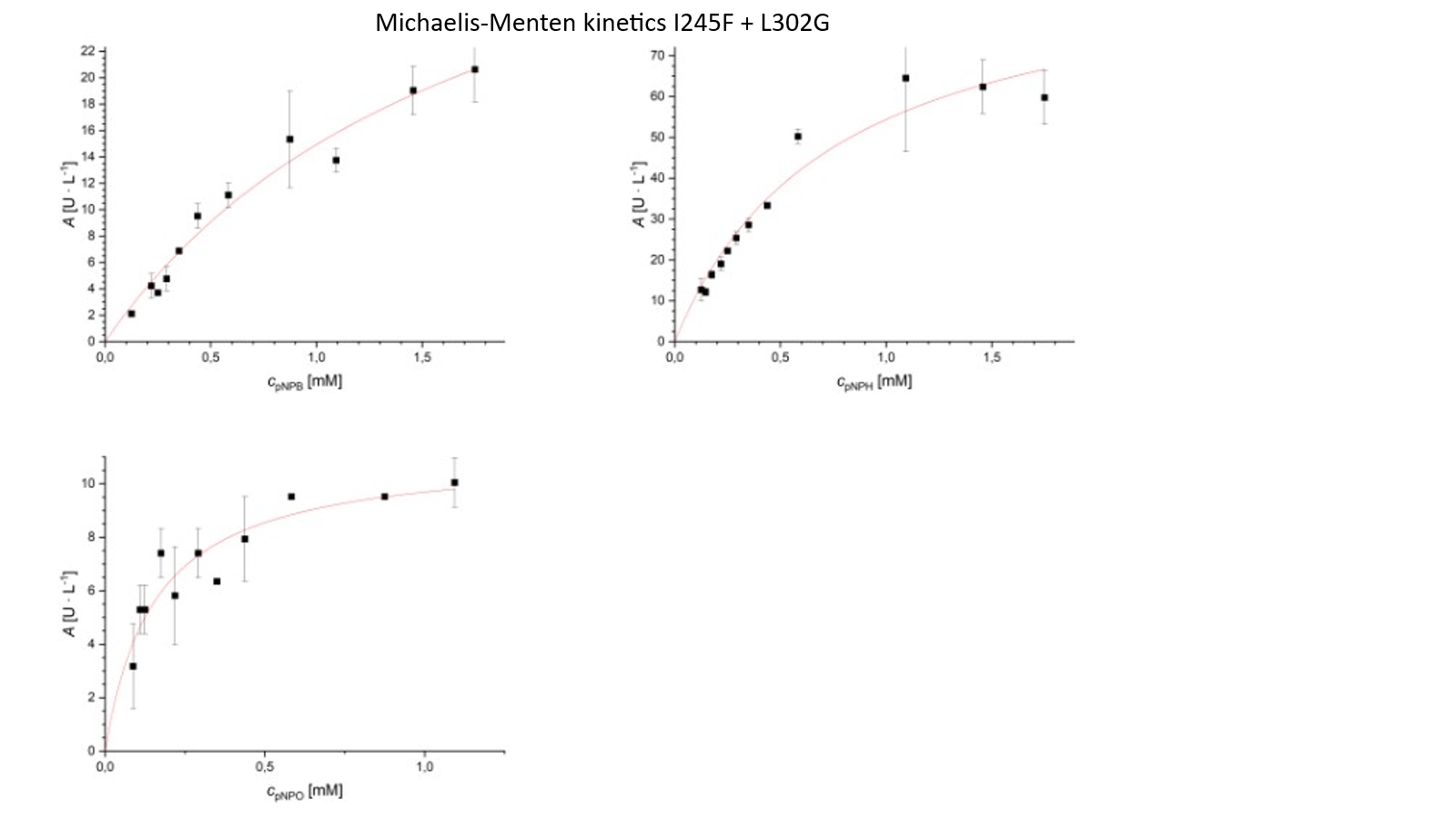


**Fig. S16.** Saturation curves of double mutant I245F+L302G against pNPB, pNPH and pNPO. Obtained data for the activity were fitted in OriginPro 2023 based on the Michaelis-Menten equation. Activity at all concentrations was measured in triplicates. Error bars represent the standard deviation.


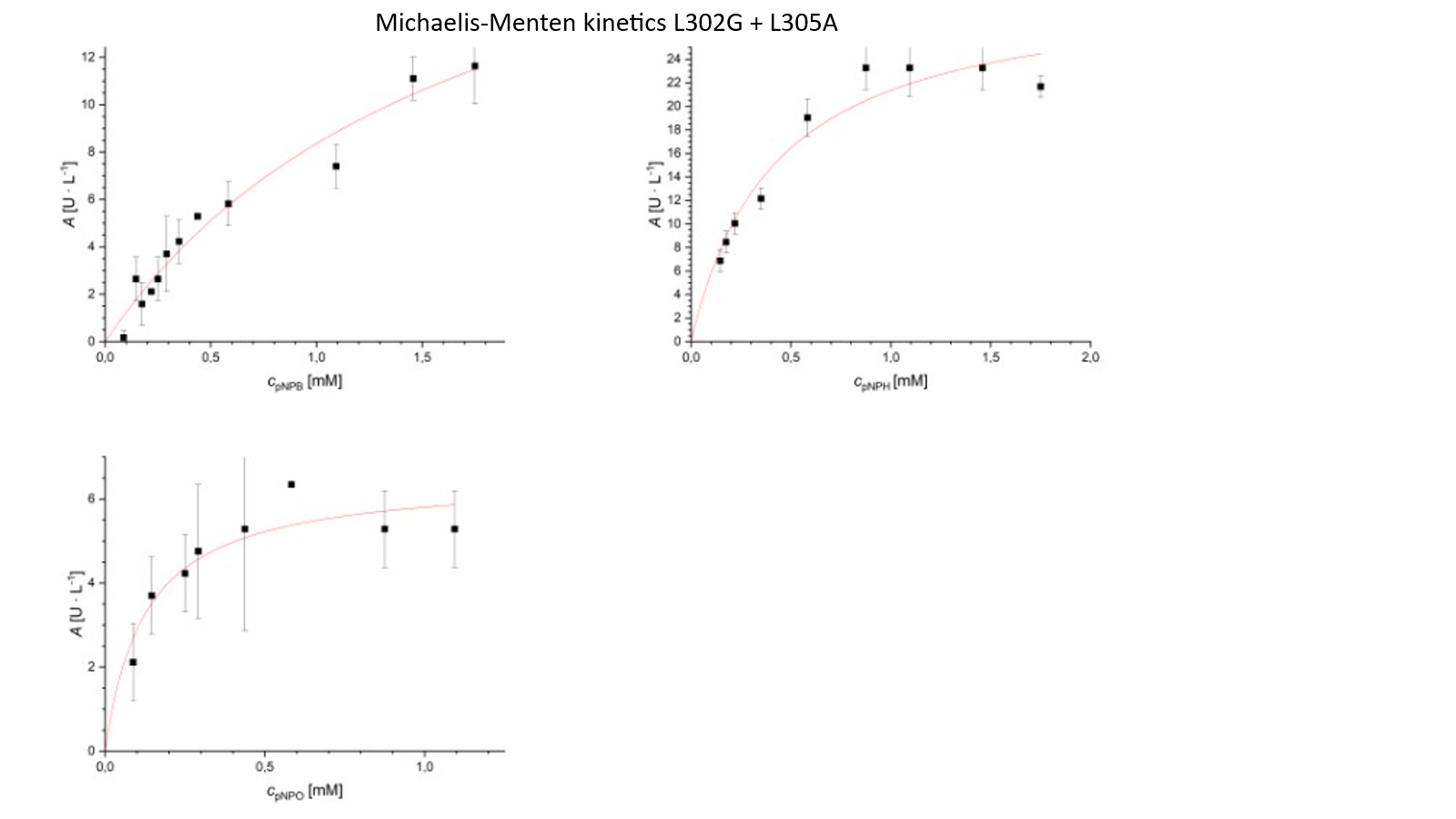


**Fig. S17.** Saturation curves of double mutant L302G+L305A against pNPB, pNPH and pNPO. Obtained data for the activity were fitted in OriginPro 2023 based on the Michaelis-Menten equation. Activity at all concentrations was measured in triplicates. Error bars represent the standard deviation.


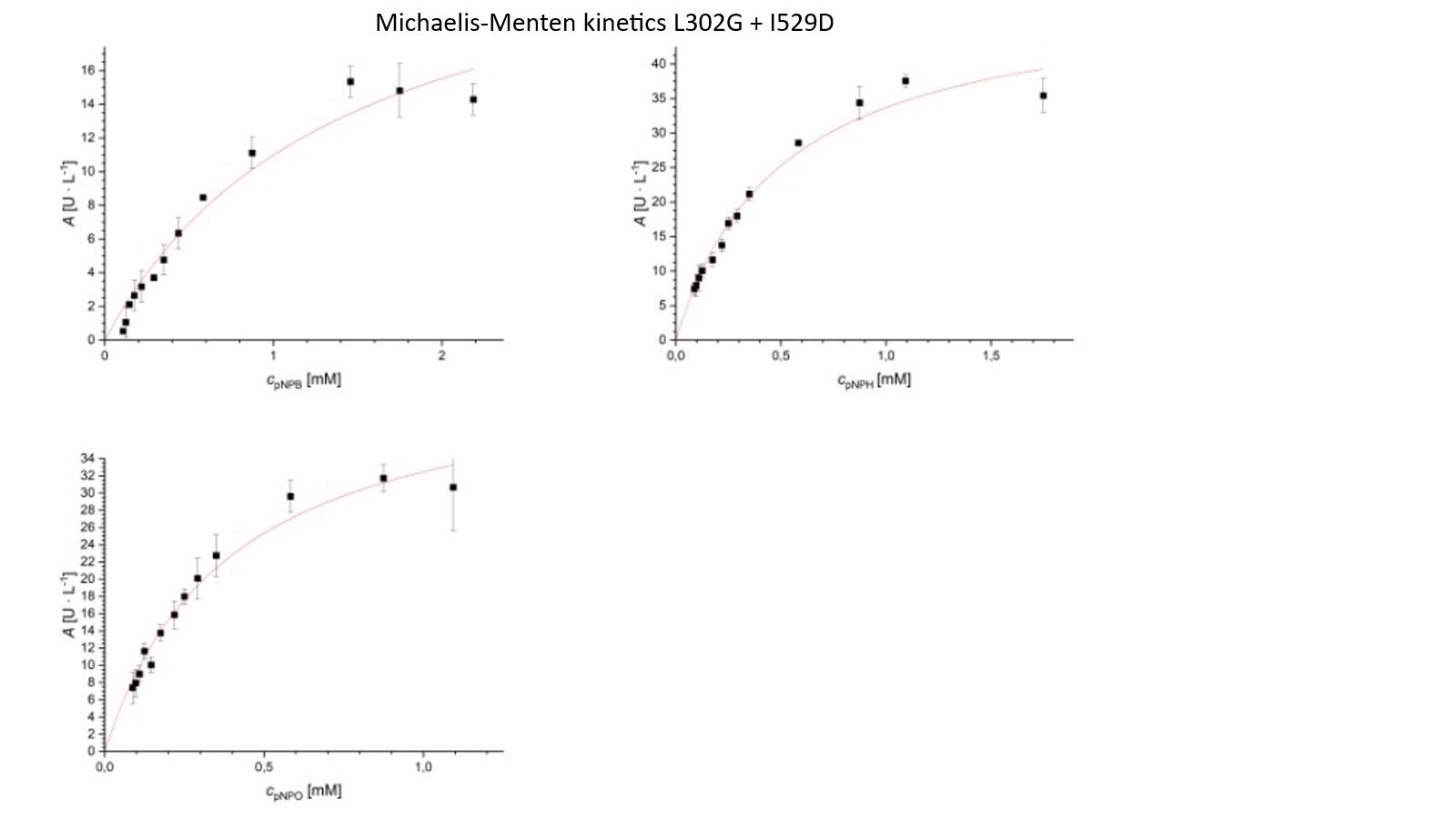


**Fig. S18.** Saturation curves of double mutant L302G+I529D against pNPB, pNPH and pNPO. Obtained data for the activity were fitted in OriginPro 2023 based on the Michaelis-Menten equation. Activity at all concentrations was measured in triplicates. Error bars represent the standard deviation.


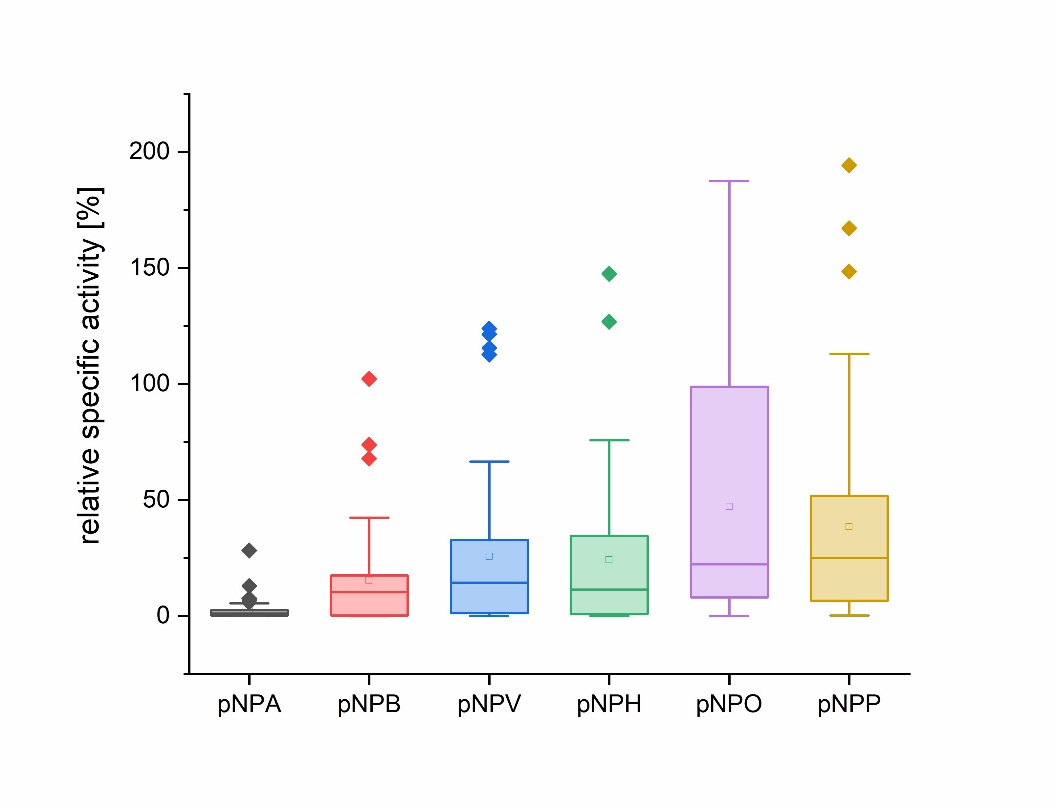


**Fig. S19.** Box plot of the statistical evaluation of photometric assays from PCI_Lip single mutants. The difference in mean values at a significance level of 0.05 is significant with a F‑value of 10.4.


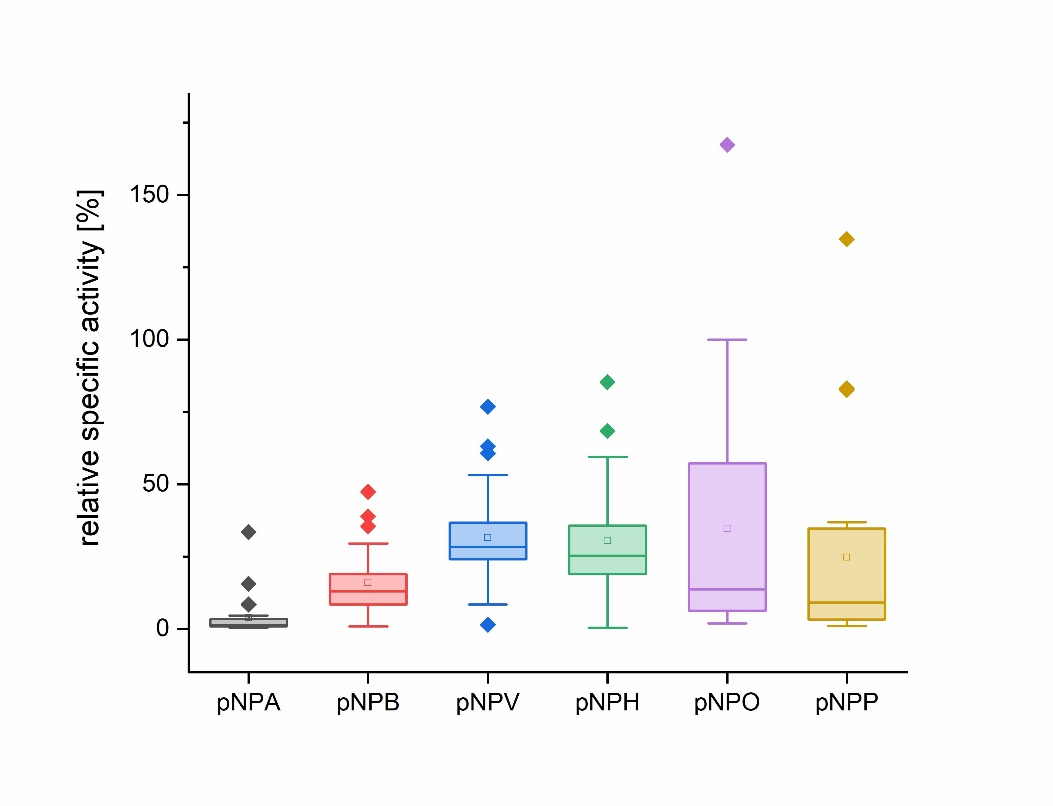


**Fig. S20.** Box plot of the statistical evaluation of photometric assays from PCI_Lip double and triple mutants. The difference in mean values at a significance level of 0.05 is significant with a F-value of 4.9.


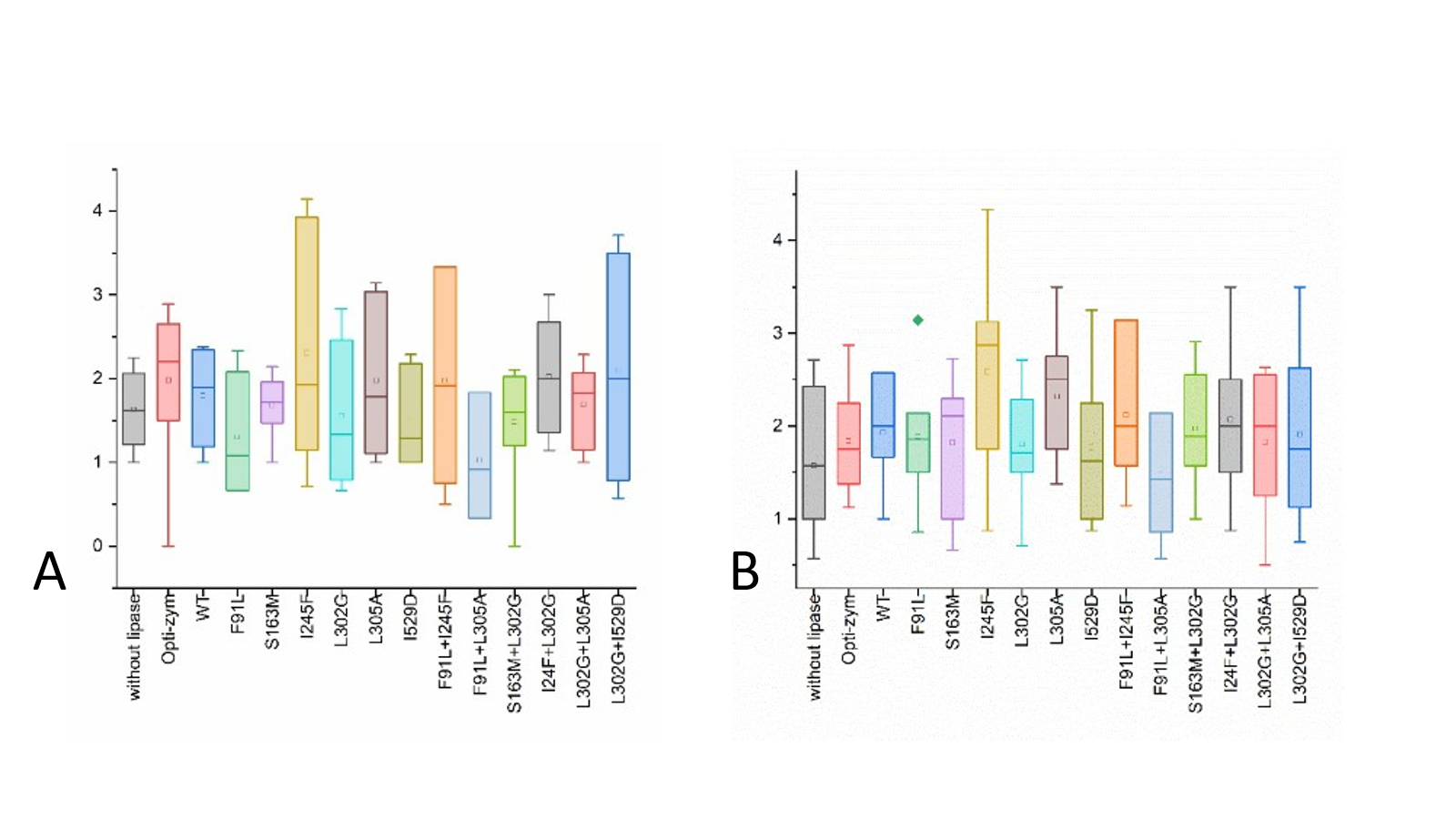


**Fig. S21.** (A) Box plot of the statistical evaluation of sensory evaluation of the smell of cheese samples prepared with PCI_Lip mutants. The difference in mean values at a significance level of 0.05 is not significant with a F-value of 0.9. (B) Box plot of the statistical evaluation of sensory evaluation of the taste of cheese samples prepared with PCI_Lip mutants. The difference in mean values at a significance level of 0.05 is not significant with a F-value of 0.9.


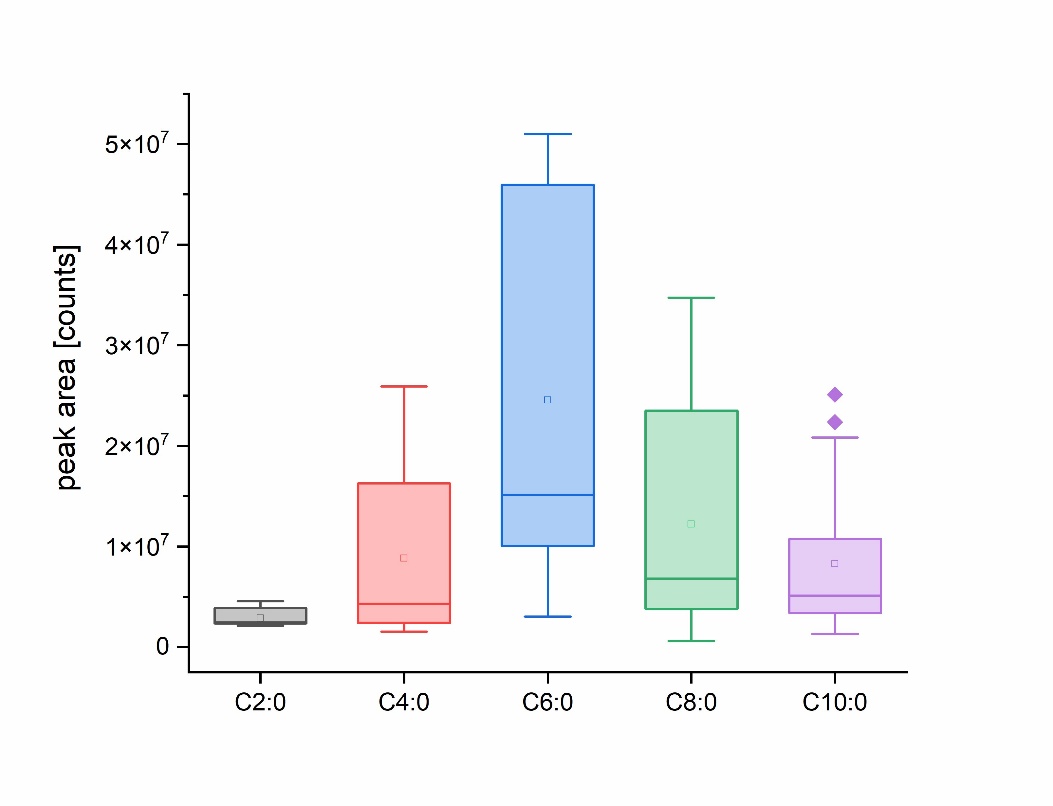


**Fig. S22.** Box plot of the statistical evaluation of the vFFA analysis of cheese samples prepared with different PCI_Lip mutants. The difference in mean values at a significance level of 0.05 is significant with a F-value of 8.1.

**Table S2**. Gene sequence of PCI_Lip WT.

|  | Gene sequence |
| --- | --- |
| PCI_Lip | ATGAACCCGCTGCCGCGTACCACCAGCGTTACCCTGGATAGCGCCAGCTTTACCGGTAGTACCATTGGTCGTGTTACCAAATTTCTGGGTATTCCGTATGCACAGCCGCCGACCGGTGACCGCCGTTTTCGCCTGCCGGCACCGATTCCGGCATATACCGGTACCGTTCGTGCCACCAGTTATGGTCCGAGCTGTCCGCAGCAGGCAGTTAGTCTGCCGCTGCCGGATGGCCTGGCAGCCGATGTGGCAGATCTGCTGGTGAATACCGTTTTTAAAGCCGTGTTTCCGGATAGTGAAGATTGTCTGACCCTGAATGTTGTTGTTCCGAATAGTGCCACCCCGACCAGCAAACTGCCGGTTGTGGTGTGGATTTTTGGCGGCGGCTTTGAACTGGGTAGTACCAGCATGTATGATGGTGGTCTGATTGTGGAACGCAGCATTCAGCTGGGTGAACCGGTTATCTATGTTAGCATGAATTATCGTCTGAGTGGTTTTGGCTTTCTGGCCAGCAAAGAAGTGAAAGATGCAGGTGTTGGCAATCTGGGTCTGCAGGATCAGCGCGAAGCACTGCGTTGGATTCAGAAATATATTGGCAATTTTGGCGGCGATCCGACCAAAGTTACCATTTGGGGCGAAAGCGCAGGCGCAATTAGTGCAGCCCTGCATATGGTGGCAAATAATGGCAATACCGAAGGCCTGTTTCGCGGTGCCTTTATGCAGAGTGGTAGCCCGATTCCGGTGGGCGATATTACCCATGGTCAGGCAACCTATGATGCAATTGTTCGCGATACCGGCTGCGCAGGTAGCAGCGATACCCTGGCATGCCTGCGCGCAGCACCGTATAGCGCCCTGAAAAATGCCATTGATAATACCCCGTTTATTTTTGATTACCAGAGCCTGGTTCTGGCATGGCTGCCGCGTACAGATGGTACCTTTCTGACCGATAATCCGCAGAGCCTGGTGCAGCAGGGTAAAGTTGCAAATGTTCCGTTTATTACCGGCGATTGCGATGATGAAGGTACCCTGTTTAGTCTGGCCAATCTGAATGTGACCACCACCAGTCAGCTGCGTACCTATCTGAAAACCATTTGGCTGCCGGGCACCACCGATACCCAGATTGATACCCTGCTGAGTCATTATCCGCTGGATCTGACCCAGGGCAGTCCGTATGGTACCGGTATTCTGAATGCCCTGAGTCCGCAGTTTAAACGTCTGGCCAGCTTTCAGGGTGACGCAGTGTTTCAGGCCCCGCGTCGCTATTTTCTGCAGCAGCGTAGTGGTAAACAGAATACCTGGGCATTTCTGAGTAAACGTTTTAAAGTTGCACCGTTTCTGGGTAGTTTTCATGCAAGCGATATTCTGAATGTTTATTTTGGCGGCGAACTGGGTGACTATCTGATTAATTTTGTTAATAAGCTGGACCCGAATGGCGCCGGCCGTGGTATTAATTGGCCGAAATATACCACCAGTAGCCCGAATCTGGTGACCTTTAATGATAATCTGCTGTTTCCGGTGACCCTGAGCCAGGATACCTTTCGCCGCGATGCCATTAATTTTCTGACCGGCCTGACCCTGGCAAATCCGATT |
| PCI_Lip without lid | ATGAAAGCCGTGTTTCCGGATAGTGAAGATTGTCTGACCCTGAATGTTGTTGTTCCGAATAGTGCCACCCCGACCAGCAAACTGCCGGTTGTGGTGTGGATTTTTGGCGGCGGCTTTGAACTGGGTAGTACCAGCATGTATGATGGTGGTCTGATTGTGGAACGCAGCATTCAGCTGGGTGAACCGGTTATCTATGTTAGCATGAATTATCGTCTGAGTGGTTTTGGCTTTCTGGCCAGCAAAGAAGTGAAAGATGCAGGTGTTGGCAATCTGGGTCTGCAGGATCAGCGCGAAGCACTGCGTTGGATTCAGAAATATATTGGCAATTTTGGCGGCGATCCGACCAAAGTTACCATTTGGGGCGAAAGCGCAGGCGCAATTAGTGCAGCCCTGCATATGGTGGCAAATAATGGCAATACCGAAGGCCTGTTTCGCGGTGCCTTTATGCAGAGTGGTAGCCCGATTCCGGTGGGCGATATTACCCATGGTCAGGCAACCTATGATGCAATTGTTCGCGATACCGGCTGCGCAGGTAGCAGCGATACCCTGGCATGCCTGCGCGCAGCACCGTATAGCGCCCTGAAAAATGCCATTGATAATACCCCGTTTATTTTTGATTACCAGAGCCTGGTTCTGGCATGGCTGCCGCGTACAGATGGTACCTTTCTGACCGATAATCCGCAGAGCCTGGTGCAGCAGGGTAAAGTTGCAAATGTTCCGTTTATTACCGGCGATTGCGATGATGAAGGTACCCTGTTTAGTCTGGCCAATCTGAATGTGACCACCACCAGTCAGCTGCGTACCTATCTGAAAACCATTTGGCTGCCGGGCACCACCGATACCCAGATTGATACCCTGCTGAGTCATTATCCGCTGGATCTGACCCAGGGCAGTCCGTATGGTACCGGTATTCTGAATGCCCTGAGTCCGCAGTTTAAACGTCTGGCCAGCTTTCAGGGTGACGCAGTGTTTCAGGCCCCGCGTCGCTATTTTCTGCAGCAGCGTAGTGGTAAACAGAATACCTGGGCATTTCTGAGTAAACGTTTTAAAGTTGCACCGTTTCTGGGTAGTTTTCATGCAAGCGATATTCTGAATGTTTATTTTGGCGGCGAACTGGGTGACTATCTGATTAATTTTGTTAATAAGCTGGACCCGAATGGCGCCGGCCGTGGTATTAATTGGCCGAAATATACCACCAGTAGCCCGAATCTGGTGACCTTTAATGATAATCTGCTGTTTCCGGTGACCCTGAGCCAGGATACCTTTCGCCGCGATGCCATTAATTTTCTGACCGGCCTGACCCTGGCAAATCCGATT |
| PCE_Lip | GAATTCATGCAGCTGGTGACCCCGAATAGCGAAGATTGTCTGACCCTGAATGTGTGGGCCCCGACCCGCCTGCCGGAAGGTACAAAAGTGCCGGTTGTTGCATGGATCTTCGGTGGCGGCTTCGAAAATGGTGGCACCAGCATGTATGATGGCGGCATTATTGTGACCCGCAGTGTGGAACTGGGCAAACCGATTGTGTATGTTAGCATGAATTATCGTGTGAGCGCACTGGGCTTCCTGGCAAGTGAAGAAGTTAAAGCAGCAGGTGTTGGTAATATTGGTCTGCAGGATCAGCGCCAGGCACTGCGTTGGATTCAGAAATATATTAGCGTGTTCAATGGTGATCCGAGTAAAGTTACCATCTGGGGCGAAAGCGCAGGCGCAATTAGCGTTGCCCTGCAGATGGTGGCCAATGGCGGTAATACCGAAGGCCTGTTCCATGCCGCCTTCATGGAAAGTGGTAGTCCGATTCCGGTGGGCGATATTACCCATGGCCAGAAATTCCATGATGCCTTCGTTGTGGCAGCAGGTTGTGAAGGTAGTAGTGATACCCTGCAGTGCCTGCGTGAAGCACCGTTCGCAACCCTGCAGCAGGCCATGGATGCAAGCCCGAGTATCTTCAGCCCGAGCAGTCTGGATCTGGCCTGGATGCCGCGTGTGGATGGCACCTTCCTGCCGGATGATCCGCAGCAGCTGGTTCTGCAGGGTAGCGTGGCCAATATTCCGTTCGTGAGCGGTGATTGTGATGATGAAGGTACCCTGTTCAGTCTGACCACCCTGAATGTTACCACCGAAGAACAGCTGCGTGATTATATTACCAGTACCTATCTGCCGGCAGCAAGCAGTAGCGATCTGGATAAACTGCTGACCCTGTATCCGGCAGATATTACCCAGGGTAGTCCGTATGATACCGGTATTCTGAATGCCCTGAGCCCGCAGTTCAAACGCCTGGCCAGCCTGCAGGGTGATCTGGTGTTCCAGGGTGCCCGTCGCTTCTTCCTGCAGCAGCAGAGCGATAAACAGAATACCTGGAGCTTCCTGAGTAAACGTCTGAAAGGCCTGCCGGGCCTGGGTAGTGTTCATGCCAGTGATCTGCTGAATATCTATGGTAGTGGCGAACTGACCGATTATCTGGTTAACTTCGTTAATAATCTGAACCCGAATGGTCCGACCGTGCCGTATTGGCCGAAATATACCACCGCCAGCGTTCAGCTGCTGACCTTCCTGGATGGCCTGGTGCCGGTGGCAATTACCACCGATACCTATCGCCAGGAAGCAATGGAGTTCATTATTCAGCTGGCCCTGGCCAATCCGCTGTAAGCGGCCGC |
| PCE_Lip with lid | AACCCGCTGCCGCGTACCACCAGCGTTACCCTGGATAGCGCCAGCTTTACCGGTAGTACCATTGGTCGTGTTACCAAATTTCTGGGTATTCCGTATGCACAGCCGCCGACCGGTGACCGCCGTTTTCGCCTGCCGGCACCGATTCCGGCATATACCGGTACCGTTCGTGCCACCAGTTATGGTCCGAGCTGTCCGCAGCAGGCAGTTAGTCTGCCGCTGCCGGATGGCCTGGCAGCCGATGTGGCAGATCTGCAGCTGGTGACCCCGAATAGCGAAGATTGTCTGACCCTGAATGTGTGGGCCCCGACCCGCCTGCCGGAAGGTACAAAAGTGCCGGTTGTTGCATGGATCTTCGGTGGCGGCTTCGAAAATGGTGGCACCAGCATGTATGATGGCGGCATTATTGTGACCCGCAGTGTGGAACTGGGCAAACCGATTGTGTATGTTAGCATGAATTATCGTGTGAGCGCACTGGGCTTCCTGGCAAGTGAAGAAGTTAAAGCAGCAGGTGTTGGTAATATTGGTCTGCAGGATCAGCGCCAGGCACTGCGTTGGATTCAGAAATATATTAGCGTGTTCAATGGTGATCCGAGTAAAGTTACCATCTGGGGCGAAAGCGCAGGCGCAATTAGCGTTGCCCTGCAGATGGTGGCCAATGGCGGTAATACCGAAGGCCTGTTCCATGCCGCCTTCATGGAAAGTGGTAGTCCGATTCCGGTGGGCGATATTACCCATGGCCAGAAATTCCATGATGCCTTCGTTGTGGCAGCAGGTTGTGAAGGTAGTAGTGATACCCTGCAGTGCCTGCGTGAAGCACCGTTCGCAACCCTGCAGCAGGCCATGGATGCAAGCCCGAGTATCTTCAGCCCGAGCAGTCTGGATCTGGCCTGGATGCCGCGTGTGGATGGCACCTTCCTGCCGGATGATCCGCAGCAGCTGGTTCTGCAGGGTAGCGTGGCCAATATTCCGTTCGTGAGCGGTGATTGTGATGATGAAGGTACCCTGTTCAGTCTGACCACCCTGAATGTTACCACCGAAGAACAGCTGCGTGATTATATTACCAGTACCTATCTGCCGGCAGCAAGCAGTAGCGATCTGGATAAACTGCTGACCCTGTATCCGGCAGATATTACCCAGGGTAGTCCGTATGATACCGGTATTCTGAATGCCCTGAGCCCGCAGTTCAAACGCCTGGCCAGCCTGCAGGGTGATCTGGTGTTCCAGGGTGCCCGTCGCTTCTTCCTGCAGCAGCAGAGCGATAAACAGAATACCTGGAGCTTCCTGAGTAAACGTCTGAAAGGCCTGCCGGGCCTGGGTAGTGTTCATGCCAGTGATCTGCTGAATATCTATGGTAGTGGCGAACTGACCGATTATCTGGTTAACTTCGTTAATAATCTGAACCCGAATGGTCCGACCGTGCCGTATTGGCCGAAATATACCACCGCCAGCGTTCAGCTGCTGACCTTCCTGGATGGCCTGGTGCCGGTGGCAATTACCACCGATACCTATCGCCAGGAAGCAATGGAGTTCATTATTCAGCTGGCCCTGGCCAATCCGCTG |

**Table S3**. Purity of the mutants expressed and purified within this work.

| Mutant | Purity [%] |
| --- | --- |
| F91M | 47.5 |
| F91Y | 71.5 |
| F129T | 79.0 |
| F129L | 71.3 |
| F129H | 71.9 |
| S163G | 53.9 |
| S163I | 58.2 |
| S163Q | 36.5 |
| I245Y | 70.2 |
| I245E | 65.3 |
| I245T | 67.3 |
| L302A | 69.4 |
| L302H | 70.6 |
| L302V | 74.6 |
| L305P | 77.1 |
| L305Q | 78.5 |
| I529H | 70.3 |
| I529R | 41.9 |
| I529T | 81.9 |
| I245F+I529D | 10.4 |
| L302G+I529D | 30.9 |
| F91L+I529D | 26.5 |
| I245F+L302G | 1.9 |
| F91L+I245F | 30.8 |
| F91L+L305A | 36.3 |
| F91L+L302G | 95.2 |
| L305A+I529D | 86.2 |
| S163M+I245F | 85.7 |
| I245F+L305A | 75.1 |
| S163M+L302G | 65.1 |
| S163M+I529D | 67.3 |
| L302G+L305A | 49.2 |
| S163M+L305A | 55.8 |
| F91L+S163M | 84.2 |
| F91L+I245F+L305A | 71.7 |
| F91L+L305A+I529D | 96.3 |
| F91L+L302G+I529D | 47.2 |
| F91L+I245F+L302G | 57.6 |
| F91L+L302G+L305A | 44.7 |
| I245F+L302G+L305A | 50.8 |
| L302G+L305A+I529D | 43.1 |
| PCE_Lip (refolded) | 74.4 |
| PCE_Lip with lid (refolded) | 96.3 |

S1.1 Protein refolding

For refolding insoluble enzymes in this work, application note 18-1134-37 AC “Rapid and efficient purification and refolding of a (histidine)_6_-tagged recombinant protein produced in *E. coli* as inclusion bodies” by GE Healthcare (Düsseldorf, Germany) was followed. *E. coli* cell pellets from 200 mL culture were resuspended in 8 mL 20 mM Tris-HCl buffer, pH 8.0. The suspension was sonicated three times for 2.5 min, 5 cycles and a power of 50% by sonifier MS72 (Bandelin). The suspension was centrifuged at 3500 x g, 4 °C for 10 min. The pellet was resuspended in 3 mL ice-cold 20 mM TRIS-HCl buffer, 0.5 M NaCl, 2 M urea, 2% triton, pH 8.0 and sonified again as described before. After centrifugation the pellet was washed with 3 mL 20 mM TRIS-HCl buffer, pH 8.0. The washed pellet was resuspended in 5 mL binding buffer (20 mM Tris-HCl, 0.5 M NaCl, 5 mM imidazole, 6 M guanidine-HCl, 1 mM *β*‑mercaptoethanol, pH 8.0) and incubated at a shaker at room temperature for 90 min. After centrifugation, the supernatant was applied to a FPLC featuring a 5 mL IMAC column with 20 mL of binding buffer. This is followed by a washing step applying 20 mL of washing buffer (20 mM TRIS-HCl, 0.5 M NaCl, 20 mM imidazole, 6 M urea, 1 mM *β*-mercaptoethanol, pH 8.0). Refolding was conducted in a linear gradient from washing buffer to refolding buffer (20 mM Tris-HCl, 0.5 M NaCl, 20 mM imidazole, 1 mM *β*-mercaptoethanol, pH 8.0) over 150 mL. Elution of the refolded protein was performed in a linear gradient from refolding buffer to elution buffer (20 mM Tris-HCl, 0.5 M NaCl, 500 mM imidazole, 1 mM *β*-mercaptoethanol, pH 8.0) for 45 mL. The whole refolding was conducted with a flow rate of 1 mL ∙ min^-1^. Refolding was followed by a desalting step conducted as described before.

S1.2 vFFA analysis of Feta-type brine cheese samples

The vFFA in the cheese samples prepared with different PCI_Lip mutants were analyzed by SPME-GC-MS. Three grams of each cheese sample were transferred to a 20 mL headspace vial (Th. Geyer) and 2 mL 2M HCl (Thermo Fisher) were added. Afterwards the mixture was homogenized carefully. All samples were prepared and measured as duplicates. Samples were subjected to a MPS 2XL multipurpose samples (GERSTEL, Mühlheim an der Ruhr, Germany), where they were incubated at 55 °C, 250 rpm for 10 min. Subsequent extraction at 55 °C, 250 rpm for 40 min was conducted with a 1 cm × 65 µm polydimethylsiloxane (PDMS)/divinylbenzene (DVB) SPME fiber (Supleco, Steinheim, Germany). For desorption of the analytes the fiber was subjected to the GC inlet at 250 °C for 90 s (SPME liner, 0.75 mm inner diameter, Supleco) and subsequently the fiber was baked out at 250 °C for 5 min. GC analysis was performed on an Agilent 7890A (Waldbronn, Germany) with a split/splitless inlet using a split ratio of 5:1). Analytes were separated on a polar VF-WAXms column (30 m × 0.25 mm, 0.25 µm film thickness, Agilent). The temperature program was as follows: 40 °C for 3 min, 5 °C ∙ min^-1^ to 240 °C for 12 min. Helium was used as carrier gas at a constant flow rate of 1.2 mL ∙ min^-1^. Detection was performed with an Agilent 5975C mass spectrometer (transferline temperature: 250 °C, source temperature: 230 °C, quadrupole temperature: 150 °C, ionization energy: 70 eV, ms scan *m/z* 33 – 300). The analytes were identified by comparing the mass spectra and retention indices to authentic standards and the National Institute of Standards and Technology (NIST) MS Search (2011).
